# Loss of the tumour suppressor LKB1/STK11 uncovers a leptin-mediated sensitivity mechanism to mitochondrial uncouplers for targeted cancer therapy

**DOI:** 10.1186/s12943-024-02061-4

**Published:** 2024-07-25

**Authors:** Andriani Angelopoulou, Giorgos Theocharous, Dimitrios Valakos, Aikaterini Polyzou, Sophia Magkouta, Vassilios Myrianthopoulos, Sophia Havaki, Marco Fiorillo, Ioanna Tremi, Konstantinos Vachlas, Theodoros Nisotakis, Dimitris-Foivos Thanos, Anastasia Pantazaki, Dimitris Kletsas, Jiri Bartek, Russell Petty, Dimitris Thanos, Rory J McCrimmon, Angelos Papaspyropoulos, Vassilis G Gorgoulis

**Affiliations:** 1 Molecular Carcinogenesis Group, Department of Histology and Embryology, Medical School, National and Kapodistrian University of Athens, Athens, Greece; 2 Biomedical Research Foundation, Academy of Athens, Athens, Greece; 3 “Thorax” Foundation - Research Center of Intensive Care and Emergency Thoracic Medicine, Athens, Greece; 4 Department of Pharmaceutical Chemistry, Faculty of Pharmacy, University of Athens, Athens, 15772 Greece; 5 Department of Pharmacy, Health and Nutritional Sciences, University of Calabria, Rende, 87036 Italy; 6 Thoracic Surgery Department, “Sotiria” Hospital, Athens, 115 27 Greece; 7 Laboratory of Biochemistry, Department of Chemistry, Aristotle University of Thessaloniki, Thessaloniki, 54124 Greece; 8 Laboratory of Cell Proliferation and Ageing, Institute of Biosciences and Applications, National Centre for Scientific Research “Demokritos”, Athens, 15341 Greece; 9 Genome Integrity Group, Danish Cancer Society Research Center, Copenhagen, 2100 Denmark; 10 Science for Life Laboratory, Division of Genome Biology, Department of Medical Biochemistry and Biophysics, Karolinska Institute, Solna, Stockholm, 171 77 Sweden; 11 Ninewells Hospital and Medical School, University of Dundee, Dundee, UK; 12 Faculty Institute for Cancer Sciences, Manchester Academic Health Sciences Centre, University of Manchester, Manchester, UK; 13 Faculty of Health and Medical Sciences, University of Surrey, Guildford, UK

**Keywords:** Non-small cell lung cancer (NSCLC), LKB1/STK11, HIF1A-LEP-UCP2 axis, Zebrafish, Metabolic stress, Piceatannol, Tyrphostin 23, Airway organoids, CRISPR/Cas9-mediated genome editing, Drug discovery

## Abstract

Non-small cell lung cancer (NSCLC) constitutes one of the deadliest and most common malignancies. The LKB1/STK11 tumour suppressor is mutated in ∼ 30% of NSCLCs, typically lung adenocarcinomas (LUAD). We implemented zebrafish and human lung organoids as synergistic platforms to pre-clinically screen for metabolic compounds selectively targeting LKB1-deficient tumours. Interestingly, two kinase inhibitors, Piceatannol and Tyrphostin 23, appeared to exert synthetic lethality with *LKB1* mutations. Although LKB1 loss alone accelerates energy expenditure, unexpectedly we find that it additionally alters regulation of the key energy homeostasis maintenance player leptin (LEP), further increasing the energetic burden and exposing a vulnerable point; acquired sensitivity to the identified compounds. We show that compound treatment stabilises Hypoxia-inducible factor 1-alpha (HIF1A) by antagonising Von Hippel-Lindau (VHL)-mediated HIF1A ubiquitination, driving *LEP* hyperactivation. Importantly, we demonstrate that sensitivity to piceatannol/tyrphostin 23 epistatically relies on a HIF1A-LEP-Uncoupling Protein 2 (UCP2) signaling axis lowering cellular energy beyond survival, in already challenged LKB1-deficient cells. Thus, we uncover a pivotal metabolic vulnerability of LKB1-deficient tumours, which may be therapeutically exploited using our identified compounds as mitochondrial uncouplers.

**Supplementary Information:**

The online version contains supplementary material available at 10.1186/s12943-024-02061-4.

## Introduction

Lung adenocarcinoma (LUAD) is the most common type of human lung cancer, falling within the spectrum of non-small cell lung cancers (NSCLCs). LUAD constitutes the leading cause of cancer-related mortality worldwide, accounting for approximately 40% of lung cancer cases [1]. Current treatment approaches for LUAD include surgical resection, chemotherapy, radiotherapy and targeted therapy, with targeted therapy offering a significant advantage in the management of patients with identifiable driver oncogenic events. However, despite the advances in lung cancer management over the last decades, prognosis remains particularly poor with a 5-year survival rate of only 15–20%, while the respective survival rate of patients with distant metastases is about 8% [1].

At the molecular level, loss of the LKB1 tumour suppressor (also known as STK11) is observed in several malignancies including, notably, over 30% of human LUADs [2]. In fact, *LKB1* is the third most commonly mutated gene in LUADs, frequently coexisting with oncogenic TP53 and KRAS [2]. LKB1 is a serine threonine kinase comprising a master regulator of the main cellular energy sensor AMP-activated Kinase (AMPK), which it activates by phosphorylation, as well as 12 other AMPK-related kinases [3]. In response to low energy conditions, such as starvation, active AMPK keeps the mammalian target of rapamycin (mTOR) at bay, preventing further cell proliferation and growth [3]. Given that *AMPK* remains genetically intact in almost all cases of tumourigenesis (∼ 99% of human tumours, Fig. S1A), it is reasonable that mTOR activation requires loss of AMPK positive regulators such as LKB1.

Nevertheless, *LKB1* tumours show a hypermetabolic phenotype, fueled by induction of the serine-glycine-one carbon pathway coupled to S-adenosylmethionine generation [4]. In zebrafish, *lkb1* mutants show defective response to metabolic stress and premature exhaustion of energy reserves [5]. Therapeutically exploiting tumour suppressor mutations is indirectly possible when they confer specific dependencies to the cancer cell. Tumour cells undergo fundamental metabolic changes to meet their increased energetic demands, frequently under nutrient-poor and hypoxic environments. Indeed, the rewired metabolism of cancer cells has been proposed to constitute the Achilles’ heel of cancer. However, whether the altered metabolic traits accompanying LKB1 loss can be exploited as a mechanism for treating *LKB1* tumours has not been so far addressed.

In this study, by implementing multiple in vivo models as well as genetically engineered or LUAD patient-derived airway organoids (AOs), we leverage the deregulated metabolic features of *LKB1* mutants in an extensive synthetic lethality screen for compounds that could selectively kill the mutants but not wild-type counterparts, and identify Piceatannol (a resveratrol analog) and Tyrphostin 23 (a tyrosine kinase inhibitor, including EGFR) as selectively lethal to *LKB1* mutants. We find that the underlying mechanism imparting sensitivity to *LKB1* tumours is based on a paradox; despite the high levels of energetic stress accompanying loss of LKB1 alone, we observe activation of the key energy homeostasis player leptin (LEP). Upon LKB1 loss, we demonstrate that *LEP* is activated at the promoter level in a HIF1A-mediated fashion, resulting in epistatic upregulation of the key mitochondrial uncoupling factor UCP2, which acts to increase energetic stress by lowering energy production. Remarkably, we find that the E3 ligase VHL which targets HIF1A for degradation is directly inhibited by our identified compounds, further stabilising HIF1A, thereby causing an overflow of the energetic burden beyond survival. Clinically, we show that the compounds indeed confer selective lethality to *LKB1* tumours, with no toxicity for LKB1-proficient cells. Taken together, we uncover an unprecedented molecular regulation connecting *LKB1* loss to leptin stabilisation, responsible for the clinically significant sensitivity to identified mitochondrial uncouplers.

## Results

### Synthetic lethality screening and transcriptome analysis reveal *leptin b* as a major Lkb1-regulated gene and uncover metabolic vulnerabilities

**Fig. 1:**
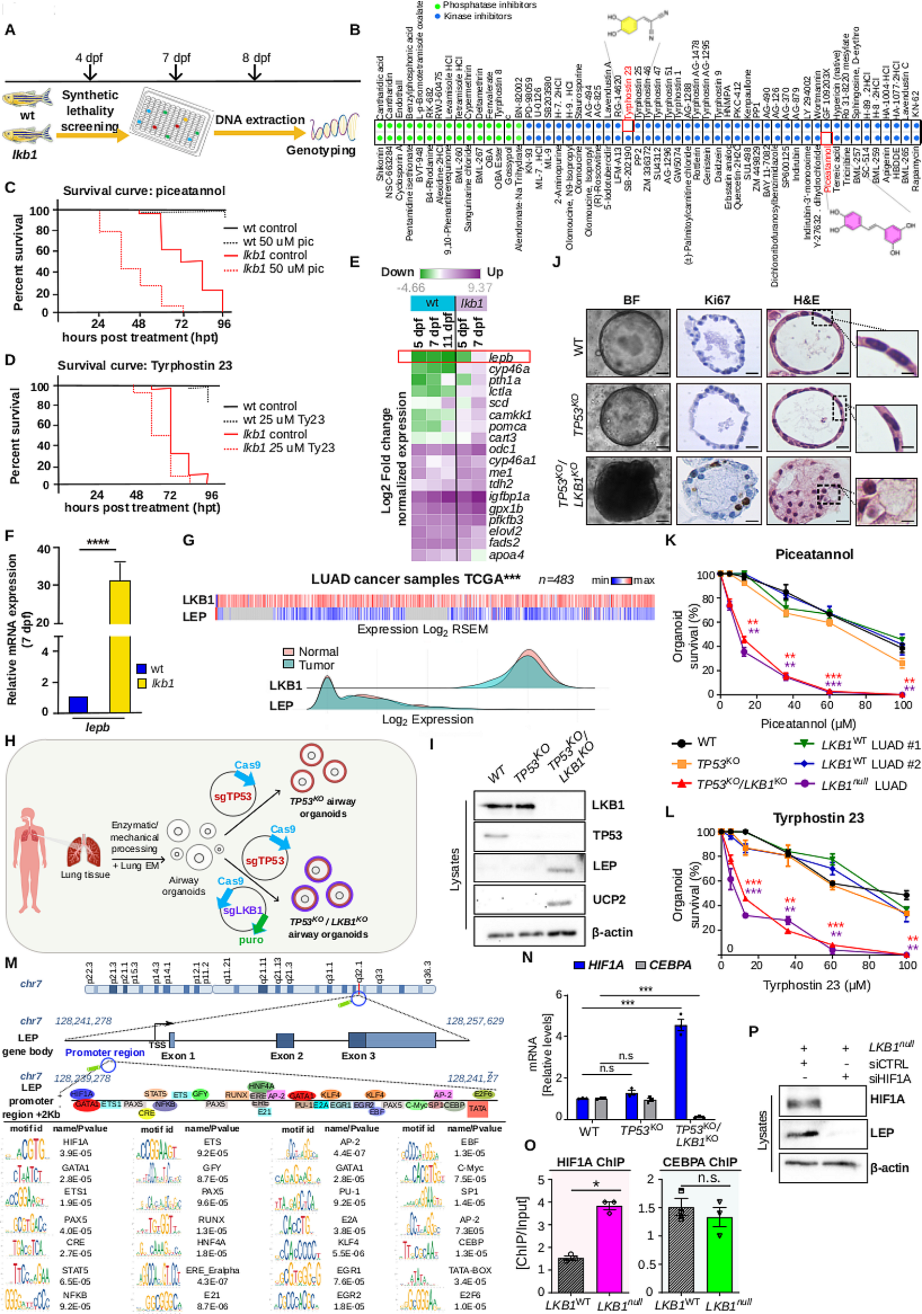
Loss of LKB1/STK11 promotes HIF1A-mediated leptin activation accompanied by selective susceptibility to metabolic activators Piceatannol and Tyrphostin 23. **(A)** Schematic depicting the timeline of the synthetic lethality screen performed on wt and *lkb1* zebrafish trunks. **(B)** Piceatannol and tyrphostin 23 were identified as synthetically lethal with loss of *lkb1* in zebrafish trunks. **(C-D)** Survival analysis of wt and *lkb1* larvae following treatment with 50 µM of piceatannol (pic), 25 µM of Tyrphostin (Ty23), or DMSO. **(C)** Pic-treated *lkb1* larvae die from 24 h post-treatment (hpt) onwards (****), while wt larvae are only slightly affected (n.s.). **(D)** Ty23- treated *lkb1* larvae die from 48 hpt onwards (****). Wt larvae are affected from 96 hpt onwards (***). ****P < 0.0001, ***P < 0.0005 compared to control treatments; Log-rank (Mantel-Cox) test. **(E)** Transcriptome analysis of total RNA isolated from wt and *lkb1* larvae at 5, 7 dpf and wt trunks at 11 dpf. **(F)** q-PCR for *lepb* mRNA levels from total RNA extracted from wt and *lkb1* trunks at 7 dpf. ****P < 0.005; two-tailed student’s t-test. **(G)** Top: heatmap showing reciprocal expression of LKB1 and LEP across LUAD tumour samples of the TCGA dataset (*n* = 483). Bottom: LKB1 and LEP expression density plots across LUAD (*n* = 483) and normal (*n* = 59) samples of the TCGA dataset. **(H)** Schematic depicting the experimental strategy implemented to acquire clonal *TP53*^*KO*^ or *TP53*^*KO*^*/LKB1*^*KO*^ AOs via CRISPR/Cas9-mediated genome editing. **(I)** Western blotting for indicated markers in AO clones. **(J)** Representative images of normal (WT), *TP53*^*KO*^ and *TP53*^*KO*^*/LKB1*^*KO*^ airway mutant organoids stained for haematoxylin and eosin (H&E) and Ki67. Scale: 10–30 μm. See also Fig. S3C. **(K)** Drug screening of indicated CRISPR/Cas9-engineered AO mutants, LUAD and WT AOs with various concentrations of piceatannol. For each concentration, stars represent statistical comparisons of *TP53*^*K*O^/*LKB1*^*KO*^ AOs or *LKB1*^*null*^ LUAD AOs (as indicated by sample color) with WT AOs. See also Additional File 3. **(L)** Same as (K), for tyrphostin 23 (Ty23). See also Additional File 3. **(M)** Human *LEP* promoter map displaying binding positions of transcription factors in sense (+) or anti-sense (-) strands potentially regulating *LEP*. Identified motifs per transcription factor are displayed, along with their P values (*P < 0.001*). See also Additional File 4. **(N)** q-PCR for HIF1A and CEBPA mRNA levels in CRISPR/Cas9-engineered AOs. **(O)** HIF1A and CEBPA ChIP on the *LEP* promoter. **(P)** Immunoblotting for indicated markers in *LKB1*^*null*^ LUAD AOs treated with siRNA against HIF1A or non-targeting siRNA (siCTRL). ***P < 0.001 and **P < 0.01, of Student’s t-test. Error bars indicate s.e.m. N.s.; non-significant. Data shown are representative of at least 3 independent experiments

We initially sought to exploit the hypermetabolic characteristics of *lkb1* zebrafish larvae to uncover vulnerabilities that could be used for targeting an Lkb1-deficient setting, such as that seen in many cancers. To that end, we performed a synthetic lethality screen aiming to identify compounds that target specifically *lkb1* mutants. We tested 80 kinase and 30 phosphatase inhibitors for their ability to selectively kill the *lkb1* larvae and not wt counterparts (Fig. 1A-B). Both the kinase and phosphatase inhibitor libraries included compounds against known targets of metabolic interest. We distributed wt, heterozygous and *lkb1* larvae at 4 days post fertilisation (dpf) in a 96-well plate (3 larvae/well) containing 10 µM of compounds. We monitored morphology and survival of the larvae for three days, since the majority of *lkb1* mutants die at 8 dpf. All surviving larvae were collected and genotyped to distinguish the *lkb1* mutants (Fig. 1A). After validation, we selected two compounds for further characterisation, Piceatannol and Tyrphostin 23, as compared to the other drugs, only those two exhibited synthetic lethality with *lkb1* larvae (Fig. 1B).

Piceatannol (3,3′, 4,5′-trans-trihydroxystilbene) is a naturally occurring, metabolically more stable analogue of resveratrol, however there is no sufficient evidence to recommend its clinical use as yet. For validation experiments, we tested a range of concentrations to identify the maximum tolerated dose. We found that *lkb1* larvae were susceptible to piceatannol treatment in a dose-dependent manner (Fig. S1B). Treatment with 50 µM of piceatannol led to death of 75% of *lkb1* larvae, 48 h post-treatment (hpt) (at 6 dpf) (Fig. 1C), while, 100% of the untreated *lkb1* larvae were still alive and only minor lethality was observed in treated wt or heterozygous (het) larvae. At 72 hpt, all piceatannol-treated *lkb1* larvae were dead and only 5% of wt/het larvae had died. These results indicate that *lkb1* larvae are exclusively susceptible to piceatannol at a concentration in which the vast majority of wt/het larvae are not affected.

Tyrphostin 23 (Ty23) is a member of the tyrphostins, which were developed as synthetic inhibitors of protein tyrosine kinases. Treatment with 25 µM Ty23 led to 50% of *lkb1* larvae dying at 60 hpt, while untreated *lkb1* larvae remained alive (Fig. 1D) and at this concentration, Ty23 was toxic to approximately 20% of wt larvae at 96 hpt. These results show that, similarly to a response to piceatannol, *lkb1* larvae are more susceptible to Ty23 than wt counterparts.

We next set out to identify factors underlying compound susceptibility, as a consequence of the hypermetabolic phenotype conferred by *lkb1* loss [5]. Depletion or interruption of the maternal nutrient supply induces gluconeogenesis to restore glucose levels, therefore by subjecting *lkb1* larvae to starvation we questioned whether glucose level modulators may be mechanistically involved in the increased drug sensitivity. By analysing the glucose metabolism transcriptome in starved *lkb1* versus wild-type larvae, we confirmed that genes associated with gluconeogenesis e.g. *pck1* and *g6pca.1* and fasting-state e.g. *pdk2b* and *idh3a*, were significantly upregulated in *lkb1* larvae (Fig. S1C). In contrast, genes associated with glycolysis, such as *pklr* and *eno4*, were expressed significantly lower in *lkb1* compared to wt larvae (Fig. S1C). Along those lines, expression of gluconeogenesis markers *pck1* and *g6pca.1* in zebrafish liver, pancreas, intestine and kidney showed that gluconeogenesis was aberrantly and prematurely activated in Lkb1-deficient larvae at 6 dpf (Fig. S1D). Interestingly, qPCR for known gluconeogenesis modulators *cremb* and *pgc1a* [6] showed that they are expressed significantly higher in *lkb1* larvae at 7 dpf (Fig. S1E). These data indicated that since the dramatic and premature increase in gluconeogenic gene expression in *lkb1* larvae precedes upregulation of the modulators, the metabolic stress reflected in a glycolysis-gluconeogenesis imbalance is most likely not mediated by *cremb* and *pgc1a*, but through other, Lkb1-regulated mechanisms.

To investigate the underlying mechanism by which Lkb1 may orchestrate metabolic adaptation conferring susceptibility to metabolic activators, we performed genome-wide transcriptional analysis of starved wt and *lkb1* trunks (Fig. 1E and S2A-B). Interestingly, the most highly upregulated gene in *lkb1* larvae at 7 dpf was *leptin b (lepb)*, which encodes for leptin, a peripheral hormone regulating feeding, glucose metabolism and energy expenditure [7]. *Lepb* (the true ortholog of mammalian leptin) was highly upregulated in *lkb1* larvae compared to wt at all the time points examined, further confirmed by qPCR for *lepb* mRNA levels (Fig. 1E-F). High leptin expression in a “starved” state is striking, as in mammals leptin levels decrease during fasting [7] and indeed, *lepb* was not upregulated in starved wt larvae at 11 dpf. These data strongly indicate that the regulation of leptin expression is Lkb1-dependent, providing a potential mechanism for LKB1-mediated metabolic adaptation.

To gain further insight into the premature lethality of *lkb1* larvae upon treatment, we analysed expression of a panel of genes involved in fasting metabolism in treated wt and *lkb1* larvae at 6 dpf. The gene panel consisted of gluconeogenesis markers (g6pca, pck1), the gluconeogenesis modulator pgc1a, known pro-inflammatory cytokines involved in this process (il1β and il6) as well as the mitochondrial uncoupler Uncoupling protein 2 (ucp2), which is also a direct downstream target of leptin [8]. The most dramatically upregulated gene in both wt and *lkb1* mutants following treatment was ucp2 (Fig. S2C-E), which upon metabolic stress and starvation, uncouples oxygen consumption from ATP synthesis [8]. Together, our data demonstrate that treatment with piceatannol or tyrphostin 23 likely acts by increasing the energetic burden over already metabolically challenged *lkb1* mutant zebrafish, possibly via leptin, thus resulting in selective lethality of *lkb1* mutants and no evident toxicity for their wt counterparts.

### *LKB1-* deficient NSCLC organoids display elevated LEP and susceptibility to compounds identified in zebrafish larvae

*LKB1* inactivating mutations rank among the most frequent genetic alterations in lung tumourigenesis and more specifically LUAD, often occurring together with *TP53* mutations [2]. Interestingly, LKB1 and LEP display opposite expression patterns across a wide panel of human LUAD tumours retrieved from the Cancer Genome Atlas (TCGA) database (Fig. 1G). To test whether loss of *LKB1* in humans also confers sensitivity to the identified compounds, we engineered normal patient-derived airway organoids (AOs) using CRISPR/Cas9 in order to obtain clonal *TP53*^*KO*^*/LKB1*^*KO*^ mutants (see Methods and Fig. 1H). The introduction of the desired frameshift mutations to AOs was verified both by sequencing and assessment of protein levels of targeted genes (Fig. 1I and S3A-B). In line with observations in zebrafish, both LEP and UCP2 were found elevated upon loss of LKB1 (Fig. 1I). Histological examination of the engineered lung organoids confirmed that *TP53*^*KO*^*/LKB1*^*K*O^ mutants recapitulated the NSCLC morphology resembling LUADwith pronounced tubule formation, loss of cellular polarity and aberrant proliferation compared to wild-type (WT) organoids or organoids carrying only *TP53*^*KO*^ mutations (Fig. 1J and S3C). To additionally assess the tumourigenic potential of *TP53*^*KO*^*/LKB1*^*KO*^ AOs, we orthotopically injected them intratracheally into the lungs of NOD-SCID mice (*n* ≥ 6), and verified successful engraftment and LUAD formation, as shown by stereoscopy, histological examination, TTF-1 (Thyroid transcription factor 1; LUAD marker) and human Keratin (KRT) staining, in more than 80% of the injected animals (Fig. S3D-E). Contrary to previous methodologies demonstrating complete lack of clonal expansion of mutated airway organoids [9], our results demonstrate that normal AOs can be engineered to obtain clonal oncogenic mutants, which are functionally capable of recapitulating the disease in vivo. As expected, although *TP53* KO alone is not sufficient to transform normal organoids, introduction of dual *TP53* and *LKB1* KOs clearly confers LUAD traits, rendering those AO mutants a valuable tool for downstream analyses. Moreover, gluconeogenesis and/or inflammation markers were significantly upregulated in the absence of LKB1 (Fig. S3F), overall indicating a state of metabolic stress conferred by LKB1 loss alone.

Apart from engineered AOs, we additionally sought to derive *LKB1*^*wt*^ and *LKB1*^*null*^ organoids from respective LUAD patients, as those organoid systems would be directly relevant at the clinical level. Indeed, LUAD AOs were derived from multiple patient biopsies and subjected to Whole Exome Sequencing (WES) or Hotspot sequencing analysis (Fig. S4A and Additional Files 1–2) to determine the *LKB1* gene status. To avoid the possibility of contamination by normal lung cells which would survive in the lung medium, LUAD AOs were generated from metastatic niches such as the lymph nodes or the liver. Alternatively, in the presence of a concurrent *TP53* mutation, LUAD AOs were cultured in the addition of the wild-type p53 stabiliser Nutlin-3a, completely eliminating noncancerous cells via p53-mediated apoptosis. As a result, two *LKB1*^*wt*^ and one *LKB1*^*null*^ LUAD AO lines were successfully established, and the *LKB1* expression status was additionally validated at the protein level (Fig. S4B-C). Of note, and in keeping with previous results, only the *LKB1*^*null*^ LUAD AO line again displayed elevated LEP and UPC2 levels (Fig. S4C).

To directly test the effects of piceatannol and tyrphostin 23 following loss of *LKB1*, we performed drug screenings on wild-type, engineered and LUAD organoids using various concentrations of the two agents and assessed cell viability via protease activity (Fig. 1K-L). In line with observations in zebrafish, piceatannol and tyrphostin 23 concentrations ranging from approximately 10–60 µM selectively arrested *LKB1*^*KO*^ mutants and *LKB1*^*null*^ organoids, resulting in approximately 40–60% reduction in survival compared to LKB1-proficient AOs (Fig. 1K-L and Additional File 3). As expected, wild-type organoids displayed no sensitivity to piceatannol treatment (Fig. 1K-L, S4D-E and Additional File 3). In order to additionally assess the impact of our metabolic activators on LKB1-deficient organoids at an intermediate concentration (36 µΜ), we evaluated the proliferative capacity of the few surviving *LKB1*^*null*^ versus *LKB1*^*wt*^ LUAD AOs. As indicated by Ki67 staining, *LKB1*^*null*^ LUAD AOs completely lost their proliferative potential in contrast to their *LKB1*^*wt*^ counterparts (Fig. S4F-G), indicating a strong anti-tumour effect even at lower lethality rates. Of note, loss of Ki67 expression in *LKB1*^*null*^-treated organoids was accompanied by induction of senescence (Fig. S5A-B), confirmed with the combined use of senolytics dasatinib and quercetin [10] (Fig. S5C) which led to subsequent elimination of senescent cells.

Apart from the selective drug-induced toxicity and aberrant increase of gluconeogenesis markers (Fig. S5D) observed in an LKB1-deficient background, we sought to gain further insight into potential disruptions of the metabolic and/or bioenergetic profile of LKB1-deficient cells, before and after treatment with the compounds. To that end, we examined differences in the organelle ultrastructure of *LKB1*^*wt*^ and *LKB1*^*null*^ LUAD AOs before and after piceatannol/tyrphostin 23 treatment, using transmission electron microscopy (TEM) (Fig. S6A). We found that although the mitochondrial structure was normal for most cells of *LKB1*^*wt*^ LUAD AOs regardless treatment, *LKB1*^*null*^ LUAD AOs mostly contained mitochondria exhibiting partial loss of cristae (Fig. S6A). Subsequent compound treatment in *LKB1*^*null*^ LUAD AOs resulted in abnormal mitochondrial elongation, widening of the cristae and acquisition of a dense matrix indicating a severely dysfunctional profile (Fig. S6A). To additionally investigate the LKB1-deficient phenotype from a functional perspective, we carried out a metabolic flux analysis to determine the oxygen consumption rate (OCR) and the extracellular acidification rate (ECAR) as a means of assessing oxidative phosphorylation and glycolysis, respectively. In keeping with our previous observations, loss of LKB1 was accompanied by decreased basal and maximal respiration and mitochondrial ATP production (Fig. S6B), while the glycolytic reserve was also decreased compared to LKB1-proficient cells (Fig. S6C). Additional treatment with either piceatannol or tyrphostin 23 further reduced both ATP production and glycolysis (Fig. S6B and C). These results provide confirmation that piceatannol and tyrphostin 23 can effectively target LKB1-deficient tumour cells in humans, by further increasing the energetic burden.

### Leptin upregulation in the absence of LKB1 is dependent on HIF1A stabilisation and recruitment on the *LEP* promoter

Leptin (LEP) is a peripheral hormone leading to increased energy expenditure [7]. As LEP was consistently found significantly elevated in all LKB1-deficient settings we examined (Fig. 1E-F, I and S4C), we set to uncover the mechanism underlying this regulation, with the aim of clarifying the molecular cause of the increased susceptibility to metabolic stressors. For that purpose, we screened the *LEP* promoter against known transcription factor motifs in order to construct a map of potential transcription factor binding sites directly regulating LEP expression, according to their position weight matrices (Fig. 1M and Additional File 4). Interestingly, 7 of the 21 identified factors (HIF1A, c-MYC, NF-kB, CEBPA, ETS, STAT5 and CRE) were already reported to directly or indirectly interact with LKB1 at various levels and in different tissues or disease settings, confirmed by an additional network analysis (Fig. S7A). In order to determine the potential relevance of these factors with LKB1 in the LUAD setting, we assessed possible expression changes using our CRISPR/Cas9-engineered AO lines. Interestingly, of all transcription factors only two displayed significant expression differences in response to LKB1 loss alone, (i) Hypoxia inducible factor-1α (HIF1A), a stress sensor mediating adaptive responses to oxidative stress and hypoxia, which has been also found upregulated via mTOR activation and increased Reactive Oxygen Species (ROS) production upon loss of LKB1 in normoxic conditions [11] and (ii) CCAAT/enhancer-binding protein alpha (CEBPA), a master regulator of hematopoiesis with a well-documented role in acute myeloid leukemia [12]; HIF1A was found increased upon loss of LKB1 compared to wild-type controls or loss of p53 alone, whereas CEBPA displayed an opposite expression pattern (Fig. 1N and S7A-B). This opposite regulation of HIF1A and CEBPA in response to LKB1 appears in line with previous evidence documenting a possibly antagonistic relationship between the two transcription factors [12]. We additionally verified these findings using our LUAD *LKB1*^*wt*^ and *LKB1*^*null*^ AOs (Fig. S7C). As direct regulation of *LEP* at the promoter level by either of the identified transcription factors has never been shown in an LKB1-dependent context, we carried out HIF1A and CEBPA chromatin immunoprecipitation (ChIP) in LUAD AOs, in order to determine the comparative affinity of each transcription factor with the *LEP* promoter in the presence or absence of LKB1. Although both HIF1A and CEBPA displayed affinity with the *LEP* promoter, only HIF1A significantly increased its affinity upon loss of LKB1, whereas CEBPA binding did not differ (Fig. 1O). Those results indicate that of the set of identified transcription factors only HIF1A likely exerts direct regulation of *LEP* transcription in an LKB1-deficient environment.

To determine the effect of HIF1A-mediated regulation of the *LEP* promoter upon loss of LKB1, we silenced HIF1A in our LUAD *LKB1*^*null*^ organoids and evaluated LEP levels. Our results demonstrated that loss of HIF1A alone was sufficient to entirely rescue LEP upregulation (Fig. 1P and S7D), thus identifying an LKB1-HIF1A-LEP signaling cascade as a predominant mechanism of LEP regulation in the LUAD context.

### Treatment with piceatannol and tyrphostin 23 inhibits HIF1A degradation by the VHL E3 ligase facilitating LEP-mediated mitochondrial uncoupling

**Fig. 2:**
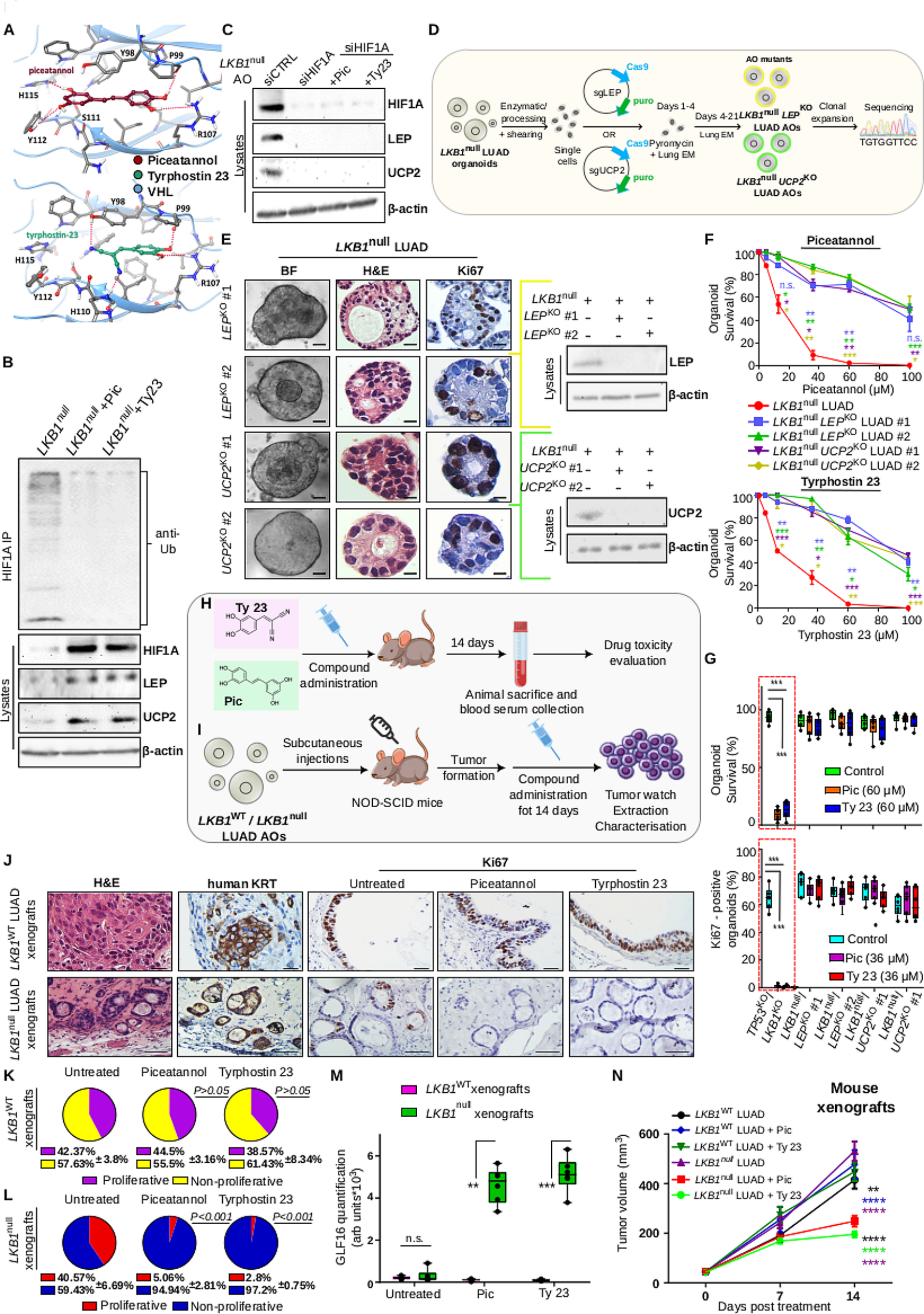
Piceatannol and Tyrphostin 23 suppress LKB1-deficient tumourigenesis in vivo by increasing the energetic burden through an epistatic HIF1A-LEP-UCP2 axis. **(A)** Docking poses of piceatannol and tyrphostin 23 in drug-binding sites of the human VHL target protein (pdb id: 6GMR and 3ZRC). Illustration of predictions of the most stable binding geometries for piceatannol (burgundy carbons) and tyrphostin 23 (green carbons). Intermolecular hydrogen bonds are depicted as dashed red lines. **(B)** In vivo ubiquitination assay in HIF1A immunoprecipitates from indicated cell lysates. Immunoprecipitates and Input lysates are probed with displayed antibodies. **(C)** Immunoblotting of AO lysates with indicated markers. **(D)** Schematic of the experimental procedure of introducing *LEP* or *UCP2* KOs in *LKB1*^*null*^ LUAD AOs. **(E)** Representative stainings and immunoblotting of two independent clones of *LKB1*^*null*^*LEP*^*KO*^ LUAD or *LKB1*^*null*^*UCP2*^*KO*^ LUAD organoids for displayed markers. See also Fig. S8 and S9A-B. Scale: 30 μm. **(F)** Drug screenings of indicated AOs with various concentrations of piceatannol/tyrphostin 23. For each concentration, stars represent statistical comparisons of *LKB1*^*null*^ LUAD AOs with the respective *LEP* or *UCP2* KO mutants (as indicated by sample color). See Additional File 3 for relevant IC50s. **(G)** Survival and proliferation of AO lines (*n* ≥ 10) under treatment or not. See also Fig. S9C. **(H)** Schematic of drug administration in mice to determine potential toxic effects. See Additional File 5. **(I) ***LKB1*^*wt*^ or *LKB1*^*null*^ LUAD AOs were subcutaneously injected in immunodeficient mice and subsequently treated with piceatannol/tyrphostin 23 to determine potential effects on tumour growth. **(J)** Immunohistochemistry in formed tumours (*n* ≥ 6). Scale: 30–60 μm. **(K-L)** Quantification of Ki67 expression levels in **(K) ***LKB1*^*wt*^ and **(L) ***LKB1*^*null*^ organoid-derived tumours having received no treatment versus treated tumours. **(M)** Quantification of GLF16 immunofluorescence in displayed mouse xenografts. See also Fig. S10. **(N)** Tumour volume curves in mice. Stars represent statistical comparisons of *LKB1*^*null*^ LUAD + Pic or *LKB1*^*null*^ LUAD + Ty23 AOs with *LKB1*^*null*^ LUAD AOs receiving no treatment or *LKB1*^*wt*^ LUAD AO counterparts, as indicated by sample color. ****P < 0.0001, ***P < 0.001, **P < 0.01, *P < 0.05 of two-way ANOVA statistical analysis. Error bars indicate s.e.m. N.s.; non-significant. Data shown are representative of at least 3 independent experiments

HIF1A-mediated LEP upregulation in an LKB1-depleted environment may augment the LUAD energetic burden via activation of downstream mitochondrial uncoupling players such as UCP2; however that regulation alone does not appear sufficient to confer lethality to *LKB1* tumours. This is evident as both zebrafish and lung organoids survive loss of LKB1 and dramatically lose viability only after treatment with the identified metabolic activators. Thus, we set to elucidate the mechanism through which piceatannol and tyrphostin 23 may further increase energetic stress beyond survival. To that end, we carried out *in silico* molecular docking simulations predicting potentially meaningful interactions between the two compounds and players of our identified signaling cascade. As our analysis did not reveal significant direct interactions of either of the drugs with HIF1A, LEP or UCP2, we looked at known regulators of these proteins. Interestingly, both piceatannol and tyrphostin 23 appeared to bind strongly with the Von Hippel-Lindau (VHL) E3 ligase, responsible for HIF1A ubiquitination and subsequent proteasomal degradation [13] (Fig. 2A). Specifically, the interface of HIF1A/VHL dimerization comprises a typical protein-protein interaction (PPI) site and ligand binding to only a part of the VHL-HIF1A PPI interface has been shown to be adequate for a measurable displacement of HIF1A [14]. Using the SZmap algorithm we carried out a computational study of solvation thermodynamics around the HIF1A recognition site of VHL which indicated a number of high-energy hydration sites in VHL, overlapping with, and possibly displaced by the two compounds, thus favoring their affinity (Fig. 2A).

As tyrphostin 23 has never been shown to interact with VHL and there is conflicting evidence regarding the ability of piceatannol to stabilise or even destabilise HIF1A, we experimentally addressed the findings of our molecular docking analysis in the LUAD setting. For that purpose, *LKB1*^*null*^ LUAD AOs were treated or not with piceatannol or tyrphostin 23 and subsequently subjected to a HIF1A ubiquitination assay (Fig. 2B and S7E). In accordance with our *in silico* observations, both drugs significantly reduced HIF1A ubiquitination levels, accompanied by robust stabilisation of total HIF1A levels as well as its downstream targets, LEP and UCP2 (Fig. 2B and S7E). To confirm that HIF1A stabilisation is indeed the direct outcome of the activity of the compounds, we silenced HIF1A in the same AO system and found that both LEP and UCP2 expression was markedly reduced, even upon piceatannol and tyrphostin 23 treatment (Fig. 2C). Taken together, our data indicate that in the absence of LKB1 both compounds may independently act to selectively stabilise HIF1A by antagonising VHL-mediated HIF1A degradation. Upon HIF1A stabilisation LEP is further upregulated, which may dramatically lower cellular energy in LUAD, potentially through UCP2 (Fig. S7F).

### LUAD organoid susceptibility to metabolic stressors is reversed upon abrogation of an epistatic LEP-UCP2 regulation

As UCP2 was found upregulated in all settings where LEP was also elevated (Fig. 1I, S2D and S4C), and further increased upon treatment with the identified metabolic stressors (Fig. 2B, S2C and S7E), we questioned whether UCP2 may indeed be responsible for the sensitivity to piceatannol and tyrphostin 23 conferred by LKB1 loss. To address this, we again utilised our *LKB1*^*null*^ LUAD AOs to introduce individual inactivating mutations via CRISPR/Cas9 targeting either *LEP* or *UCP2* (Fig. 2D). Validation of successful *LEP* and *UCP2* KOs in all clonal mutant organoids was performed by sequencing as well as Western blotting (Fig. 2E, S8 and S9A-B). To avoid bias, at least two clonal organoid lines carrying either *LEP*^*KO*^ (*LKB1*^*null*^*LEP*^*KO*^ LUAD AO #1 and #2) or *UCP2*^*KO*^ (*LKB1*^*null*^*UCP2*^*KO*^ LUAD AO #1 and #2) mutations were selected for downstream analyses. Introduction of the mutations did not result in significant morphological alterations in the parental LUAD AO line, and the LUAD phenotype was maintained (Fig. 2E).

Although treatment of *LKB1*^*null*^ LUAD AOs with piceatannol or tyrphostin 23 led again to increased cell death, loss of LEP was sufficient to reverse sensitivity to drugs, in both organoid clones (Fig. 2F and Additional File 3). The same outcome was observed in the absence of UCP2 (Fig. 2F and Additional File 3), indicating that sensitivity to compounds is indeed mediated through LEP and its downstream target UCP2. In support of that, while all LKB1-deficient AOs with an intact LEP-UCP2 axis display lethality in response to high concentrations of the compounds or lose their proliferative capacity in milder concentrations (Fig. 2G, S4D-G and S9C), additional loss of LEP or UCP2 results in a complete rescue of lethality or compromised proliferation (Fig. 2G and S9C). Those results indicate that the increased metabolic imbalance due to LKB1 loss is mechanistically mediated through activation of LEP-UCP2 signaling, which is substantially enhanced by the identified metabolic stressors. Upon abrogation of LEP-UCP2 signaling, the compounds become no longer efficient.

### Metabolic stressors piceatannol and tyrphostin 23 suppress human LKB1-deficient LUAD organoid xenografts in vivo

To evaluate the translatability of the therapeutic potential of the tested compounds in vivo, we initially treated mice with piceatannol or tyrphostin 23, and then proceeded to assess prospective toxic effects (Fig. 2H). Previous research has shown that intravenous administration of piceatannol at a concentration of 100 mg/kg of body weight was sufficient for the drug to exert its metabolic activity, without considerable toxic effects [15]. As intravenous administration in small animals such as NOD-SCID mice is not easily feasible, and in order to achieve maximum drug bioavailability, we chose to administer intraperitoneally 100 mg piceatannol/kg of body weight once a day, over a minimum period of 14 days. Regarding tyrphostins, oral administration has been found more effective compared to the intraperitoneal route [16]. Given the lack of available information about in vivo administration of tyrphostin 23, and taking into account the similar molecular weight and activity to piceatannol, we administered *per os* 100 mg tyrphostin 23/kg of body weight once a day, over the same time period as piceatannol. Treatment of mice (*n* ≥ 4) with piceatannol or tyrphostin 23 did not lead to significant changes in any critical clinical parameter, including major biochemical/toxicity markers compared to control mice (Additional File 5). Additionally, all mice survived the treatment (100% viability) and displayed no significant body weight alterations (Additional File 5).

We next set out to investigate the effect of the compounds on tumour growth in vivo (Fig. 2I). To test this, *LKB1*^*wt*^ and *LKB1*^*null*^ LUAD AOs were subcutaneously injected in NOD-SCID mice (*n* ≥ 6) until palpable tumours were formed. Successful engraftment was achieved for both organoid lines (Fig. 2J). Of note, Ki67 stainings in excised tumours demonstrated decreased proliferation only in *LKB1*^*null*^ samples having received treatment, accompanied by induction of senescence (Fig. 2K-M and S10). Importantly, and in line with our previous findings, treatment of mice with piceatannol or tyrphostin 23 resulted in a statistically significant decrease in tumour volume, only in the absence of LKB1 (60–70% reduction; Fig. 2N). These results indicate that treatment of LKB1-deficient LUAD tumours with piceatannol or tyrphostin 23 may constitute a productive and tolerable therapeutic strategy for human patients.

## Discussion

By exploiting the hyperactivated metabolism of *lkb1* mutants to uncover vulnerabilities specific to cells/tissues harbouring *LKB1* mutations, in an unbiased synthetic lethality screen we identified piceatannol and tyrphostin 23 as compounds selectively killing the *lkb1* mutants, leaving their wt counterparts largely unharmed. Through the synergistic implementation of NSCLC patient-derived airway organoids, we additionally elucidate the molecular mechanism through which the tumour suppressor LKB1 may orchestrate metabolic adaptation in LUAD, offering a unique opportunity for meaningful therapeutic interventions. We demonstrate that sensitivity of *LKB1* mutants to the identified compounds is achieved through a two-step process, gradually increasing energetic stress and lowering available energy reservoirs. In the first step, loss of LKB1 leads to significant upregulation of leptin and its downstream target UCP2 which reduces cellular energy, in a HIF1A-mediated fashion (Fig. S11). As *LKB1* mutants have profoundly low ATP levels [5], it is conceivable that further decrease in ATP production upon targeted treatment may be deleterious. Indeed, in the second phase of our model, additional treatment with piceatannol or tyrphostin 23 elicits mitochondrial uncoupling by substantially amplifying the effect of the HIF1A-LEP-UCP2 axis, resulting in cellular senescence or cell death (Fig. S11). Moreover, abrogation of HIF1A, LEP or UCP2 expression is sufficient to reverse sensitivity to both compounds, overall indicating that an epistatic regulation in place is responsible for the observed synthetic lethality, in LKB1-deficient settings. Of critical importance, piceatannol and tyrphostin 23-mediated toxicity appears significantly limited on wild-type organoids and mouse models, rendering both metabolic activators promising clinical targets against *LKB1*-deficient tumours.

Our work demonstrates that dual induction of *TP53*^*KO*^ and *LKB1*^*KO*^ mutations is sufficient to drive LUAD transformation, in accordance with published literature identifying co-occurrence of *TP53* and *LKB1* mutations, without *KRAS* mutations, in 10% of LUADs [2]. Importantly, our methodology led to acquisition of clonal *TP53*^*KO*^*/LKB1*^*KO*^ human AO mutants, as well as clonal *LKB1*^*null*^*LEP*^*KO*^ and *LKB1*^*null*^*UCP2*^*KO*^ LUAD AO mutants, overcoming previously reported obstacles in acquiring clonal mutations in AOs [9]. It is possible that concurrent introduction of *TP53* with other potent tumour suppressor mutations (such as *LKB1*) in wild-type AOs may lead to higher clonal growth rates.

Our data demonstrate that along with the evident piceatannol- and tyrphostin 23-mediated cell death observed both ex vivo and in vivo, cellular senescence is additionally induced. Senescence is a stress-response mechanism leading to cell growth arrest as a consequence of DNA damage [10]. This is a finding of high importance, as accumulating genomic instability may drive tumour cells to escape from senescence and acquire considerably more aggressive features and harmful paracrine activity, ultimately increasing the overall tumour burden. As senescent cells are progressively becoming therapeutically targetable due to the emerging field of senolytics, strategies eliminating senescent cells may be additionally implemented for complete eradication of LKB1-deficient tumours following treatment with metabolic stressors. In our experimental setting, piceatannol- or tyrphostin 23-mediated senescence was eradicated by simultaneous administration of dasatinib and quercetin, a known senolytic scheme (D + Q) [10]. Thus, combinatorial treatment schemes based on simultaneous administration of our identified metabolic stressors together with such senolytics in the context of LKB1-null lung cancer may constitute a highly efficient approach against cancer relapse.

## Conclusions

In conclusion, our work elucidates the molecular mechanism underlying a critical metabolic vulnerability of LKB1-deficient cells, which can be readily exploited against *LKB1* tumourigenesis. By using precision medicine model systems, we unravel a novel signaling pathway conferring susceptibility to identified mitochondrial uncouplers and elimination of the *LKB1* tumour burden without substantial toxicity.

## Electronic Additional file

Below is the link to the electronic Additional file.

**Additional file 1**: Hotspot sequencing verifying the exact location of desired mutations in *TP53* and *LKB1* genes in *LKB1*^*null*^ LUAD airway organoids.

**Additional file 2**: Whole exome sequencing (WES) in *LKB1*^*wt*^ LUAD airway organoids #1 and #2.

**Additional file 3**: IC50 values for all organoid lines subjected to drug screenings in Figs. 1K-L and 2F.

**Additional file 4**: Transcription factor motif scanning on the *LEP* promoter.

**Additional file 5**: Blood test, survival and body weight change results corresponding to toxicity testing of piceatannol and tyrphostin 23 in mice.

**Additional file 6**: **Supplementary Fig. 1**. Lkb1-regulated mechanisms and not typical gluconeogenesis modulators are responsible for premature gluconeogenesis in lkb1 larvae. (A) Doughnut chart displaying the *AMPK* genomic mutation profile across all cancer samples of the TCGA database. *AMPK* remains mutation-free in the vast majority (∼ 99%) of samples, indicating that loss of AMPK may be lethal. AMPK function may be exclusively modulated by known regulators such as LKB1. (B) Survival analysis of wt and *lkb1* larvae after treatment with 25 µM, 50 µM piceatannol (pic) or vehicle. Larvae were treated at 4 dpf and monitored until 8 dpf. Treatment of *lkb1* larvae with piceatannol results in premature death of *lkb1* larvae from 24 hpt onwards in a dose-dependent manner. * *P*-value < 0.05, **** *P*-value < 0.001, n.s. *P*-value not significant; (*lkb1* 25 µM *, *lkb1* 50 µM****, wt 25/50 μM n.s.). All *P*-values were calculated in comparison to control treatments and calculated with Log-rank (Mantel-Cox) test. (C) Gene expression analysis of total RNA in wt and *lkb1* trunks at 6 dpf, using a Zebrafish Glucose metabolism PCR array. Genes associated with ‘low energy levels’ and gluconeogenesis (*pck1*, *g6pca.1*, *idh3a* and *pdk2b*) are upregulated. Genes associated with glycolysis (*eno4* and *pklr*) are downregulated. Data represent two independent experiments (10 trunks of larvae/sample). Differential expression was set at a log2 fold change of > 1.5 or <-1.5. (D) Gene expression analysis from total RNA extracted from *lkb1* zebrafish trunks at 5–7 dpf, and wt trunks at 5–11 dpf to assess expression of gluconeogenesis markers *pck1*, *g6pca1*, and (E) gluconeogenesis regulators *cremb* and *pgc1a*. The upregulation of *pck1* and *g6pca1* at 6 dpf, precedes upregulation of the regulators *cremb* and *pgc1* at 7 dpf. Data represent the means, ± standard errors of the means (SEM) and are pooled from three independent experiments. *** *P*-value < 0.002, **** *P*-value < 0.0001; two-way ANOVA with Sidak’s multiple comparisons test.

**Additional file 7**: **Supplementary Fig. 2**. Transcriptome analysis reveals Lkb1-regulated expression patterns. Transcriptome analysis of total RNA isolated from wt and *lkb1* larvae at 5, 7 dpf and wt trunks at 11 dpf. Heatmap of (A) regulatory genes and (B) non-regulatory genes associated with key cellular processes. Data represents fold change of log2 rpm. (C) Piceatannol and Tyrphostin 23 treatment leads to significant upregulation of *ucp2* expression. q-PCR for indicated marker mRNA levels in total RNA extracted from wt and *lkb1* trunks at 6 dpf. *Pgc1*a does not show differential expression upon treatment. *Pck1* is marginally upregulated in wt larvae upon treatment, while *g6pca* is downregulated in treated *lkb1* larvae. *Ucp2* is highly upregulated in both wt and *lkb1* samples upon treatment. Data represent the means, ± standard errors of the means (SEM) and are pooled from three independent experiments. (D) q-PCR analysis of *ucp2* in wild-type (wt) and *lkb1* larvae during development. The maternal nutrient supply is depleted at 5 dpf. *Ucp2* expression is increased in wt larvae only when they are under severe metabolic stress (after 8 dpf). In contrast, *ucp2* is already expressed earlier and at much higher levels in the *lkb1* mutants. Data represent the means, ± standard errors of the means (SEM) and are pooled from three independent experiments. *** *P*-value < 0.002, **** *P*-value < 0.0001; two-way ANOVA with Sidak’s multiple comparisons test. (E) Gene expression analysis of *il6* and *il1β* in total RNA extracted from wt and *lkb1* trunks at 7 dpf. Both genes were significantly upregulated in *lkb1* samples. Data represent the means, ± standard errors of the means (SEM) and are pooled from three independent experiments. * *P*-value < 0.02; two-tailed student’s t-test. Dpf: days post fertilisation.

**Additional file 8**: **Supplementary Fig. 3**. *TP53*^*KO*^*/LKB1*^*KO*^ airway organoids exhibit features of lung adenocarcinoma in vitro and in vivo. (A-B) PCR amplification products of CRISPR/Cas9-mutated *TP53* (A) or *LKB1* (B) were obtained using primers flanking the targeted exons (exon 3 and 1, respectively). PCR products were subjected to TA cloning into a pGEM-T vector and subsequent sequencing revealed indels at the expected locations. For each sgRNA used, the targeted alleles are displayed. PAM sequences are underlined in red. In order to capture extensive genomic rearrangements the BLAT pairwise sequence alignment algorithm was used, whereas less extensive rearrangements are depicted using the sangerseq_viewer Python package. (C) Quantification of Ki67-positive cells in Fig. 1J demonstrating that *TP53*^*KO*^*/LKB1*^*KO*^ organoids assume a highly proliferative phenotype, in accordance with their LUAD morphology. (D) *TP53*^*KO*^*/LKB1*^*KO*^ organoids were orthotopically injected in the lungs of NOD-SCID mice (*n* ≥ 6). Stereoscopic lung tumour images, H&E, Thyroid Transcription Factor 1 (TTF-1) and human Keratin stainings verify successful engraftment of *TP53*^*KO*^*/LKB1*^*KO*^ AO cells growing as LUADs following intratracheal injections. Scale: 20–100 μm. White arrows point to areas of tumour growth. (E) Successful engraftment and subsequent tumour growth was observed in ≥ 80% of mice injected with *TP53*^*KO*^*/LKB1*^*KO*^ AOs. (F) q-PCR data displaying comparative mRNA levels of indicated genes in WT or CRISPR-Cas9-engineered *TP53*^*KO*^ and *TP53*^*KO*^*/LKB1*^*KO*^ AOs. Among the upregulated genes are markers of gluconeogenesis (*PCK1*, *PPARGC1*), the gluconeogenesis and cAMP responsive element modulator *CREM*, as well as established inflammation markers (*IL6*, *IL1B*). ***P < 0.001 and **P < 0.01, of Student’s t-test. Error bars indicate s.e.m. Data shown are representative of at least 3 independent experiments.

**Additional file 9**: **Supplementary Fig. 4**. LEP is upregulated in *TP53*^*KO*^*/LKB1*^*KO*^ and *LKB1*^*null*^ LUAD organoids accompanied by enhanced sensitivity to identified metabolic activators. (A) LUAD AOs were generated after surgical resection of tumour tissue from LUAD patients and subsequently subjected to thorough genetic and histological characterisation. (B) Representative images of human LUAD organoids stained for haematoxylin and eosin (H&E) and Ki67. Two LKB1-proficient and one LKB1-null LUAD organoid lines were utilized (*LKB1*^*wt*^ LUAD AO #1/#2 and *LKB1*^*null*^ LUAD AO, respectively). Scale: 30 μm. (C) Western blotting from lysates of all LUAD AOs in (B) displaying upregulated LEP and UCP2 in *LKB1*^*null*^ LUAD AOs. (D-E) Representative bright-field (BF) images and survival assessment of WT, *LKB1*^*wt*^ or *LKB1*^*null*^ LUAD organoid cultures receiving or not treatment with (D) tyrphostin 23 (60 µΜ) or (E) piceatannol for 5 days. Only *LKB1*^*null*^ AOs die at the end of the treatment. Scale: 60 μm. (F-G) Immunohistochemistry on *LKB1*^*wt*^ and *LKB1*^*null*^ LUAD AOs to assess Ki67 levels upon treatment or not with (F) tyrphostin 23 or (G) piceatannol, at a less lethal dose (36 µΜ). *LKB1*^*null*^ AOs display complete loss of proliferative capacity upon treatment. ***P < 0.001, of Student’s t-test. Error bars indicate s.e.m. N.s.; non-significant. Data shown are representative of at least 3 independent experiments.

**Additional file 10**: **Supplementary Fig. 5**. Treatment of *LKB1*^*null*^ LUAD organoids with metabolic stressors induces senescence. (A) Gating parameters on *LKB1*^*wt*^ and *LKB1*^*null*^ samples for FACS experiments. (B) FACS plots and quantification of the percentage of senescent cells upon treatment of *LKB1*^*wt*^ or *LKB1*^*null*^ LUAD organoids with piceatannol or tyrphostin 23 at sublethal concentrations (36 µM) versus untreated counterparts. Senescence was assessed by implementing the rapid senescence detection fluorophore-conjugated GLF16 compound [10]. Treatment of organoids with either metabolic stressors led to significant induction of senescence compared to control only in LKB1-null conditions. (C) FACS plots and relevant quantification confirming that combined use of senolytics dasatinib and quercetin (D + Q) on *LKB1*^*null*^ LUAD organoids receiving treatment with piceatannol or tyrphostin 23 significantly eradicates senescent cell populations. Piceatannol/tyrphostin 23-mediated growth arrest is achieved even at low concentrations, via senescence induction. (D) mRNA levels of gluconeogenesis genes in *LKB1*^*wt*^ and *LKB1*^*null*^ AOs. The gluconeogenesis regulator *PDK2* is found dramatically increased in the absence of LKB1. ***P < 0.001 and **P < 0.01, of Student’s t-test. Error bars indicate s.e.m. N.s.; non-significant. Data shown are representative of at least 3 independent experiments.

**Additional file 11**: **Supplementary Fig. 6**. LKB1 deficiency is accompanied by a deregulated mitochondrial and bioenergetic profile exacerbated by piceatannol or tyrphostin 23 treatment. (A) Transmission electron microscopy (TEM) pictures of indicated organoid lines before and after treatment with piceatannol or tyrphostin 23 (36 µM). WT AOs were used as reference for normal mitochondrial structure, indicated with single black arrow. Most cells of *LKB1*^*wt*^ LUAD AOs displayed normal mitochondria, with or without treatment. Most cells of *LKB1*^*null*^ LUAD AOs exhibited aberrant mitochondrial structure, further deteriorated upon piceatannol or tyrphostin 23 treatment. Double black arrows indicate aberrant mitochondrial morphology, such as partial loss of cristae, cristae widening and mitochondrial elongation. N: nucleus; Scale bar: 1000 nm. (B) Oxygen Consumption Rate (OCR) of indicated untreated or piceatannol/tyrphostin 23-treated cells was determined using the Seahorse XFe96, via metabolic flux analysis. *LKB1*^*null*^ LUAD cells display a decrease in both basal and maximal respiration, as well as mitochondrial ATP production, compared to *LKB1*^*wt*^ LUAD counterparts, and the effect is exacerbated upon compound treatment. (C) Extracellular Acidification Rate (ECAR) of indicated untreated or piceatannol/tyrphostin 23-treated cells was determined as in (B). *LKB1*^*null*^ LUAD cells show a decrease in glycolytic reserve, which is further reduced upon compound treatment. Data represent the % average ± SD over *LKB1*^*wt*^ LUAD cells, *n* = 3. Two-way ANOVA, ns = not significant, *P < 0.05, **P < 0.005, ***P < 0.0005, ****P < 0.00005.

**Additional file 12**: **Supplementary Fig. 7**. LKB1-dependent changes in transcription factor levels identified as potential regulators of the LEP gene. (A) STRING network representing the functional relationship between LKB1 and the potentially *LEP*-regulating transcription factors identified in Fig. 1M. (B) q-PCRs of indicated genes in CRISPR/Cas9-engineered AOs displaying either non-significant changes between conditions (WT, *p53*^*KO*^, *p53*^*KO*^*/LKB1*^*KO*^ AOs) or expression changes likely not attributed to LKB1 alone. (C) Western blotting and q-PCR for indicated markers in *LKB1*^*wt*^ and *LKB1*^*null*^ LUAD AOs. Western blotting differences are verified among multiple independent experiments (*n* ≥ 5). (D) Densitometry for Western blotting in Fig. 1P. (E) Quantification of ubiquitination levels across different conditions in Fig. 2B. (F) Schematic depicting the identified LKB1-dependent regulation of *LEP* via HIF1A, in LKB1-proficient and -deficient LUADs. In the presence of LKB1, low expression of HIF1A results in lack of HIF1A-mediated activation of *LEP* transcription, with a considerable impact on overall LEP levels. In contrast, upon loss of LKB1, upregulation of HIF1A leads to high-affinity *LEP* promoter occupancy by HIF1A, resulting in significantly increased LEP levels. Additional treatment with piceatannol or tyrphostin 23 further stabilises HIF1A via inhibition of VHL-mediated HIF1A ubiquitination, resulting in hyperactivation of LEP. ***P < 0.001, of Student’s t-test. Error bars indicate s.e.m. N.s.; non-significant. Data shown are representative of at least 3 independent experiments.

**Additional file 13**: **Supplementary Fig. 8**. Sequencing results verifying successful *LEP* KO in *LKB1*^*null*^ LUAD organoids. (A-B) PCR amplification products of the mutated alleles were obtained using primers flanking the targeted exons. PCR products were subjected to TA cloning into a pGEM-T vector and subsequent sequencing revealed indels at the expected locations. For each sgRNA used, the WT sequences and the targeted alleles are displayed. PAM sequences are underlined in red. In order to capture extensive genomic rearrangements the BLAT pairwise sequence alignment algorithm was used, whereas less extensive rearrangements are depicted using the sangerseq_viewer Python package. When the BLAT algorithm is used, nucleotides marked in blue are successfully aligned between WT and mutant alleles, whereas lower case nucleotides in black indicate the introduction of an indel.

**Additional file 14**: **Supplementary Fig. 9**. Sequencing results verifying successful *UCP2* KO in *LKB1*^*null*^ LUAD organoids. (A-B) PCR amplification products of the mutated alleles were obtained using primers flanking the targeted exons. PCR products were subjected to TA cloning into a pGEM-T vector and subsequent sequencing revealed indels at the expected locations. For each sgRNA used, the WT sequences and the targeted alleles are displayed. PAM sequences are underlined in red. In order to capture extensive genomic rearrangements the BLAT pairwise sequence alignment algorithm was used, whereas less extensive rearrangements are depicted using the sangerseq_viewer Python package. When the BLAT algorithm is used, nucleotides marked in blue are successfully aligned between WT and mutant alleles, whereas lower case nucleotides in black indicate the introduction of an indel. (C) Representative bright-field (BF) images and Ki67 stainings of the indicated AO lines receiving or not treatment with piceatannol or tyrphostin 23 for 5 days. See also Fig. 2G for respective quantification Scale: 30–60 μm.

**Additional file 15**: **Supplementary Fig. 10**. Piceatannol and Tyrphostin 23 exclusively suppress transplanted LKB1-deficient human LUAD organoids in vivo. GLF16 immunofluorescence in untreated, piceatannol- or tyrphostin 23-treated *LKB1*^*wt*^ and *LKB1*^*null*^ mouse xenografts. Treatment with either compound induces in vivo senescence only in *LKB1*^*null*^ mouse xenografts. Scale: 30 μm. Quantification is presented in Fig. 2M.

**Additional file 16**: **Supplementary Fig. 11**. Model depicting a biphasic escalation of energetic stress in human LKB1-null LUAD and tumour elimination strategy. Upon loss of LKB1 in lung cancer, cells already enter a state of severe metabolic stress, characterised by a marked imbalance between glycolysis and gluconeogenesis and acquisition of a hypermetabolic phenotype leading to accelerated energy expenditure. However, *LEP* is additionally upregulated by LKB1 loss at the promoter level through HIF1A stabilisation, resulting in activation of a HIF1A-UCP2 signaling axis culminating in partial uncoupling of oxidative phosphorylation from ATP production, which reduces cellular energy reservoirs, contributing to metabolic stress. Additional treatment of LKB1-deficient cells with metabolic activators piceatannol and tyrphostin 23 further exacerbates the effects of the HIF1A-UCP2 axis, thus selectively conferring senescence-driven growth arrest or lethality to LKB1-deficient cells, via metabolic exhaustion above a sustainable threshold. In contrast, LKB1-proficient tumours fail to sufficiently upregulate the HIF1A-UCP2 axis, rendering tumour cells incapable of acquiring susceptibility to metabolic activators.

**Additional file 17**

## Data Availability

No datasets were generated or analysed during the current study.

## Abbreviations

AMPK: AMP-activated Kinase
AOs: Airway organoids
BME: Basement Membrane Extract
CEBPA: CCAAT/enhancer-binding protein alpha
ChIP: Chromatin immunoprecipitation
Dpf: Days post fertilisation
ECAR: Extracellular Acidification Rate
EGFR: Epidermal Growth Factor Receptor
FFPE: Formalin-fixed paraffin embedded
Het: Heterozygous
HIF1A: Hypoxia-inducible factor 1-alpha
Hpt: Hours post-treatment
KO: Knock-out
KRT: Keratin
LEP: Leptin
LKB1: Liver Kinase B1
LUAD: Lung adenocarcinoma
mTOR: Mammalian target of rapamycin
NOD-SCID: Non-obese diabetic-severe combined immunodeficiency disease mice
NSCLC: Non-small cell lung cancer
OCR: Oxygen Consumption Rate
PPI: Protein-protein interaction
ROS: Reactive Oxygen Species
STK11: Serine/Threonine Kinase 11
TCA: Cycle Tricarboxylic Acid Cycle
TEM: Transmission Electron Microscopy
TTF-1: Thyroid transcription factor 1
UCP2: Uncoupling Protein 2
VHL: Von Hippel-Lindau
WES: Whole Exome Sequencing
WT: Wild-Type
