# Supplementary figures and images for: Loss of the tumour suppressor LKB1/STK11 uncovers a leptin-mediated sensitivity mechanism to mitochondrial uncouplers for targeted cancer therapy
Source: Mol Cancer. 2024 Jul 25;23:147. doi: 10.1186/s12943-024-02061-4 (PMC11270803; doi:10.1186/s12943-024-02061-4)

Figure S1

A

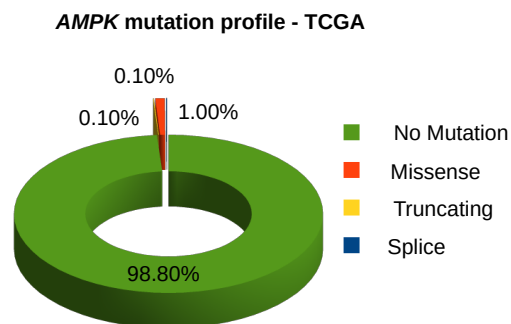

B

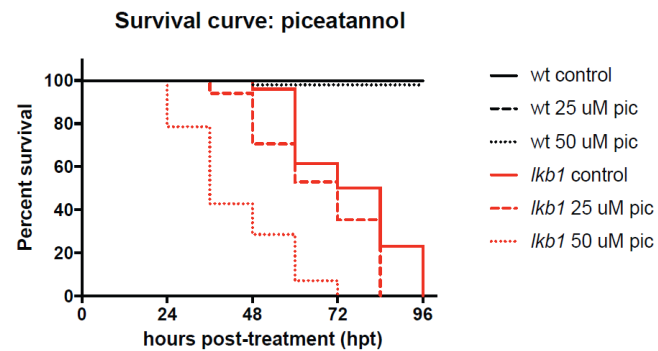

C

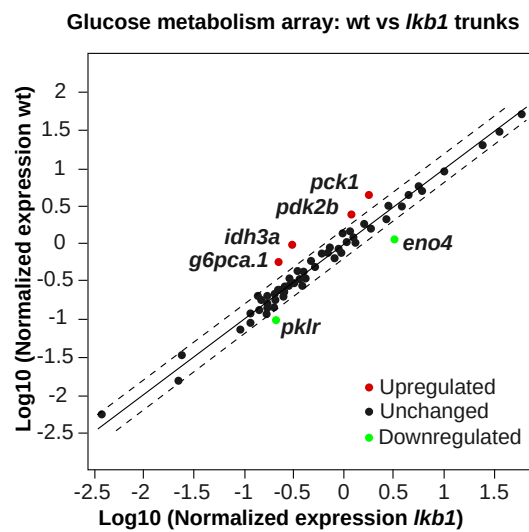

D

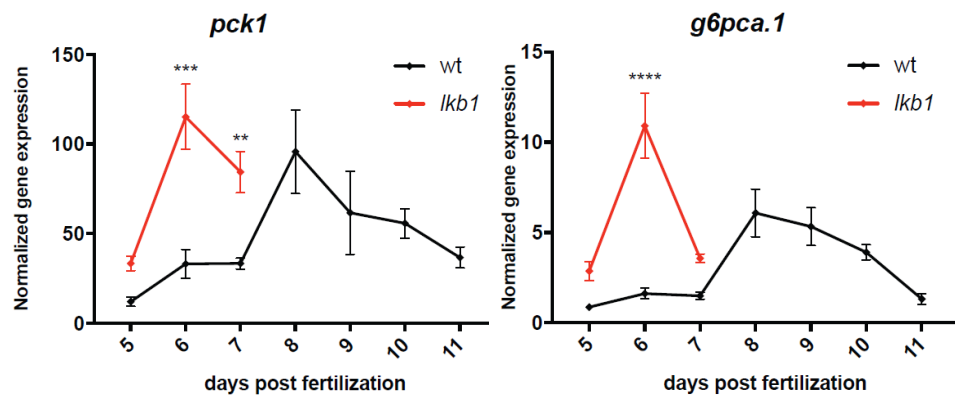

E

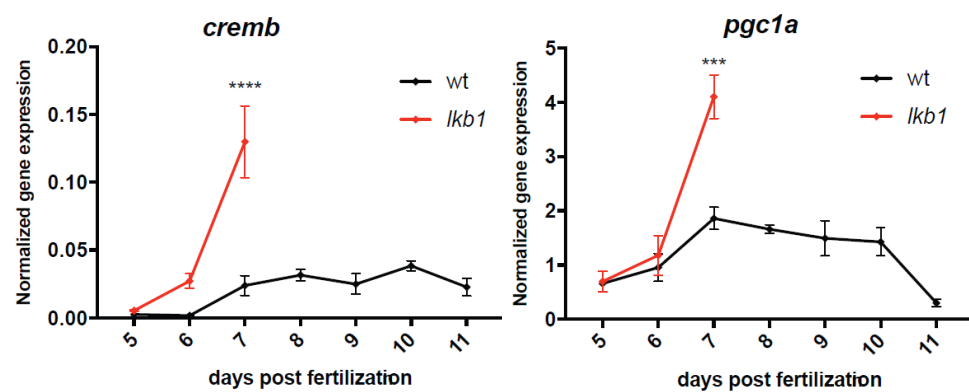

Supplement: Supplementary file 6 — Additional file 6: Supplementary Fig. 1. Lkb1-regulated mechanisms and not typical gluconeogenesis modulators are responsible for premature gluconeogenesis in lkb1 larvae. (A) Doughnut chart displaying the AMPK genomic mutation profile across all cancer samples of the TCGA database. AMPK remains mutation-free in the vast majority (∼ 99%) of samples, indicating that loss of AMPK may be lethal. AMPK function may be exclusively modulated by known regulators such as LKB1. (B) Survival analysis of wt and lkb1 larvae after treatment with 25 µM, 50 µM piceatannol (pic) or vehicle. Larvae were treated at 4 dpf and monitored until 8 dpf. Treatment of lkb1 larvae with piceatannol results in premature death of lkb1 larvae from 24 hpt onwards in a dose-dependent manner. * P-value < 0.05, **** P-value < 0.001, n.s. P-value not significant; (lkb1 25 µM *, lkb1 50 µM****, wt 25/50 μM n.s.). All P-values were calculated in comparison to control treatments and calculated with Log-rank (Mantel-Cox) test. (C) Gene expression analysis of total RNA in wt and lkb1 trunks at 6 dpf, using a Zebrafish Glucose metabolism PCR array. Genes associated with ‘low energy levels’ and gluconeogenesis (pck1, g6pca.1, idh3a and pdk2b) are upregulated. Genes associated with glycolysis (eno4 and pklr) are downregulated. Data represent two independent experiments (10 trunks of larvae/sample). Differential expression was set at a log2 fold change of > 1.5 or <-1.5. (D) Gene expression analysis from total RNA extracted from lkb1 zebrafish trunks at 5–7 dpf, and wt trunks at 5–11 dpf to assess expression of gluconeogenesis markers pck1, g6pca1, and (E) gluconeogenesis regulators cremb and pgc1a. The upregulation of pck1 and g6pca1 at 6 dpf, precedes upregulation of the regulators cremb and pgc1 at 7 dpf. Data represent the means, ± standard errors of the means (SEM) and are pooled from three independent experiments. *** P-value < 0.002, **** P-value < 0.0001; two-way ANOVA with Sidak’s multiple comparisons [file 12943_2024_2061_MOESM6_ESM.pdf]

Figure S2

A

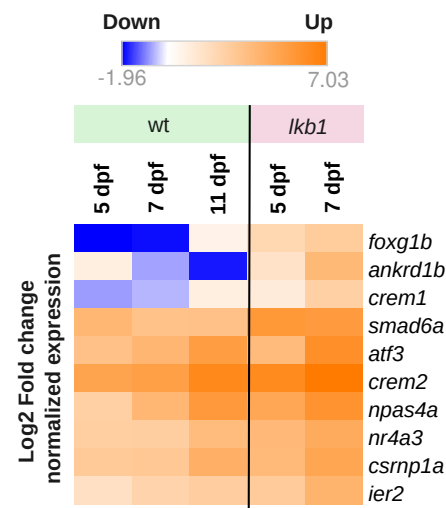

B

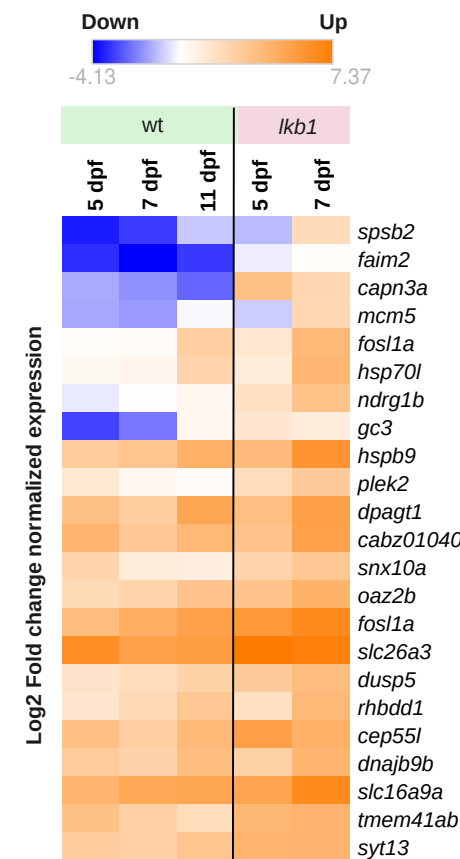

C

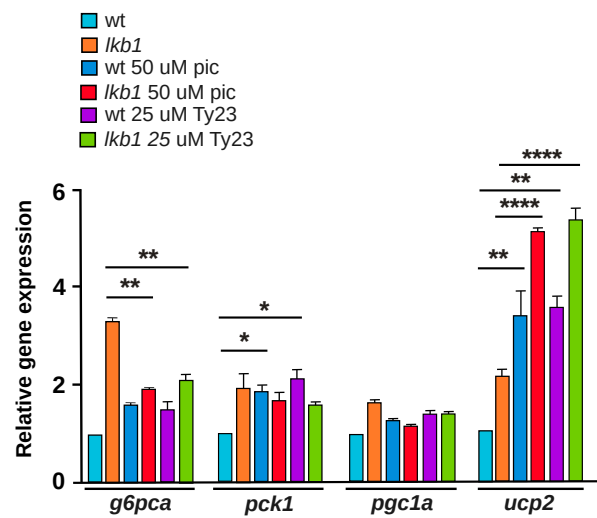

D

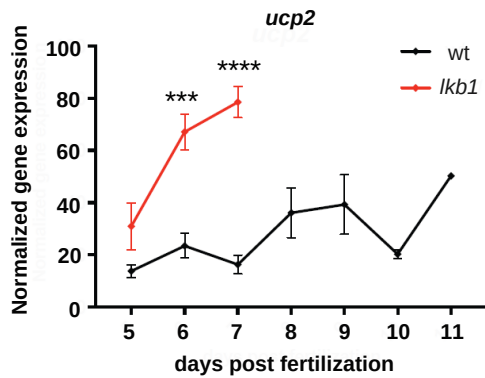

E

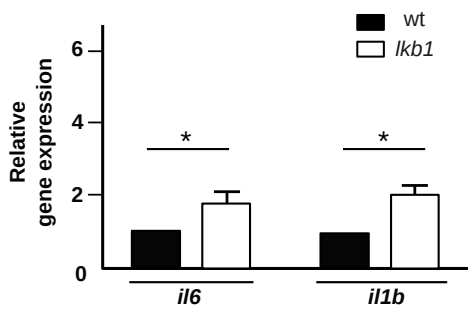

Supplement: Supplementary file 7 — Additional file 7: Supplementary Fig. 2. Transcriptome analysis reveals Lkb1-regulated expression patterns. Transcriptome analysis of total RNA isolated from wt and lkb1 larvae at 5, 7 dpf and wt trunks at 11 dpf. Heatmap of (A) regulatory genes and (B) non-regulatory genes associated with key cellular processes. Data represents fold change of log2 rpm. (C) Piceatannol and Tyrphostin 23 treatment leads to significant upregulation of ucp2 expression. q-PCR for indicated marker mRNA levels in total RNA extracted from wt and lkb1 trunks at 6 dpf. Pgc1a does not show differential expression upon treatment. Pck1 is marginally upregulated in wt larvae upon treatment, while g6pca is downregulated in treated lkb1 larvae. Ucp2 is highly upregulated in both wt and lkb1 samples upon treatment. Data represent the means, ± standard errors of the means (SEM) and are pooled from three independent experiments. (D) q-PCR analysis of ucp2 in wild-type (wt) and lkb1 larvae during development. The maternal nutrient supply is depleted at 5 dpf. Ucp2 expression is increased in wt larvae only when they are under severe metabolic stress (after 8 dpf). In contrast, ucp2 is already expressed earlier and at much higher levels in the lkb1 mutants. Data represent the means, ± standard errors of the means (SEM) and are pooled from three independent experiments. *** P-value < 0.002, **** P-value < 0.0001; two-way ANOVA with Sidak’s multiple comparisons test. (E) Gene expression analysis of il6 and il1β in total RNA extracted from wt and lkb1 trunks at 7 dpf. Both genes were significantly upregulated in lkb1 samples. Data represent the means, ± standard errors of the means (SEM) and are pooled from three independent experiments. * P-value < 0.02; two-tailed student’s t-test. Dpf: days post fertilisation. [file 12943_2024_2061_MOESM7_ESM.pdf]

Figure S3

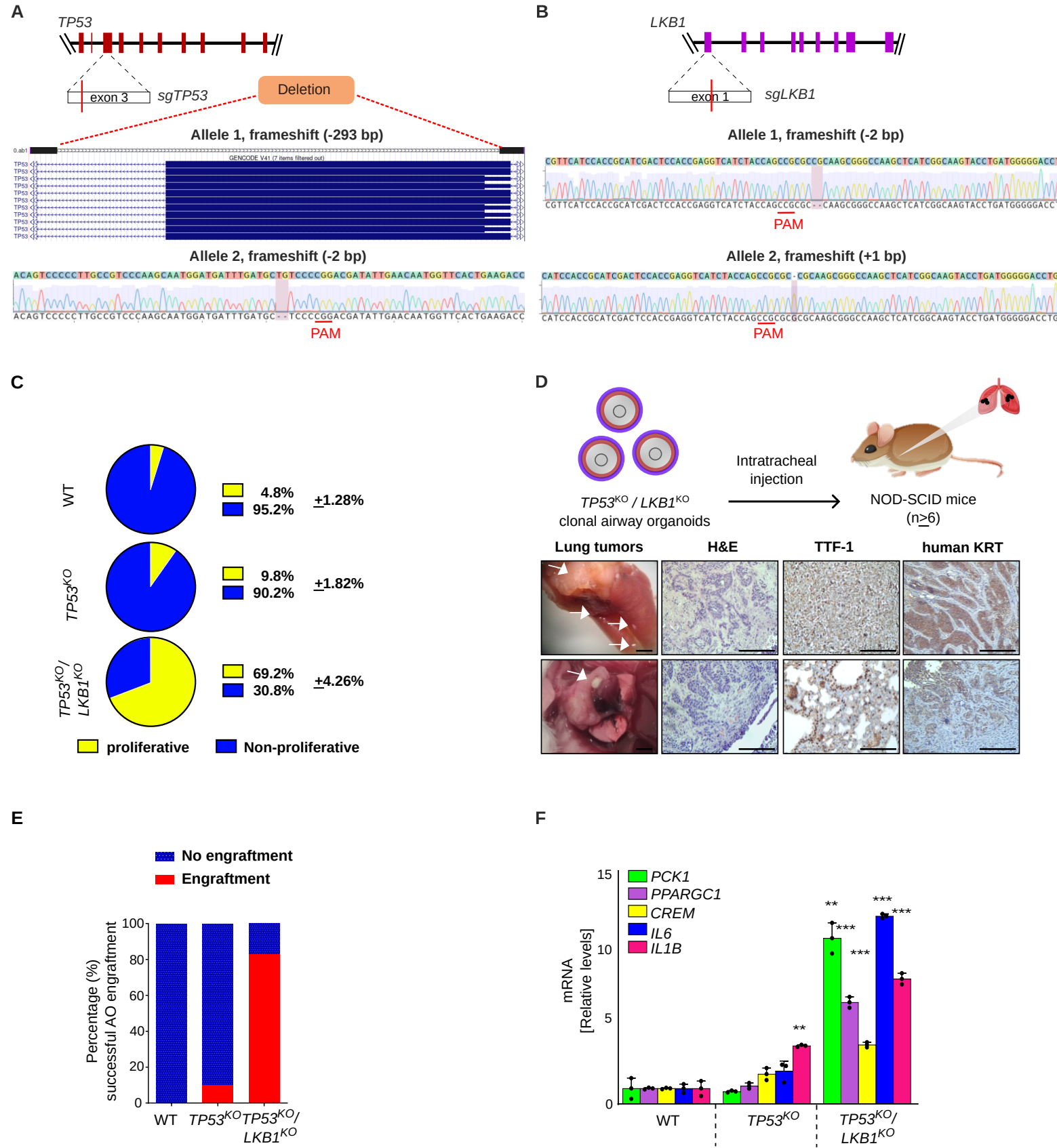

Supplement: Supplementary file 8 — Additional file 8: Supplementary Fig. 3. TP53KO/LKB1KO airway organoids exhibit features of lung adenocarcinoma in vitro and in vivo. (A-B) PCR amplification products of CRISPR/Cas9-mutated TP53 (A) or LKB1 (B) were obtained using primers flanking the targeted exons (exon 3 and 1, respectively). PCR products were subjected to TA cloning into a pGEM-T vector and subsequent sequencing revealed indels at the expected locations. For each sgRNA used, the targeted alleles are displayed. PAM sequences are underlined in red. In order to capture extensive genomic rearrangements the BLAT pairwise sequence alignment algorithm was used, whereas less extensive rearrangements are depicted using the sangerseq_viewer Python package. (C) Quantification of Ki67-positive cells in Fig. 1J demonstrating that TP53KO/LKB1KO organoids assume a highly proliferative phenotype, in accordance with their LUAD morphology. (D) TP53KO/LKB1KO organoids were orthotopically injected in the lungs of NOD-SCID mice (n ≥ 6). Stereoscopic lung tumour images, H&E, Thyroid Transcription Factor 1 (TTF-1) and human Keratin stainings verify successful engraftment of TP53KO/LKB1KO AO cells growing as LUADs following intratracheal injections. Scale: 20–100 μm. White arrows point to areas of tumour growth. (E) Successful engraftment and subsequent tumour growth was observed in ≥ 80% of mice injected with TP53KO/LKB1KO AOs. (F) q-PCR data displaying comparative mRNA levels of indicated genes in WT or CRISPR-Cas9-engineered TP53KO and TP53KO/LKB1KO AOs. Among the upregulated genes are markers of gluconeogenesis (PCK1, PPARGC1), the gluconeogenesis and cAMP responsive element modulator CREM, as well as established inflammation markers (IL6, IL1B). ***P < 0.001 and **P < 0.01, of Student’s t-test. Error bars indicate s.e.m. Data shown are representative of at least 3 independent experiments. [file 12943_2024_2061_MOESM8_ESM.pdf]

Figure S4

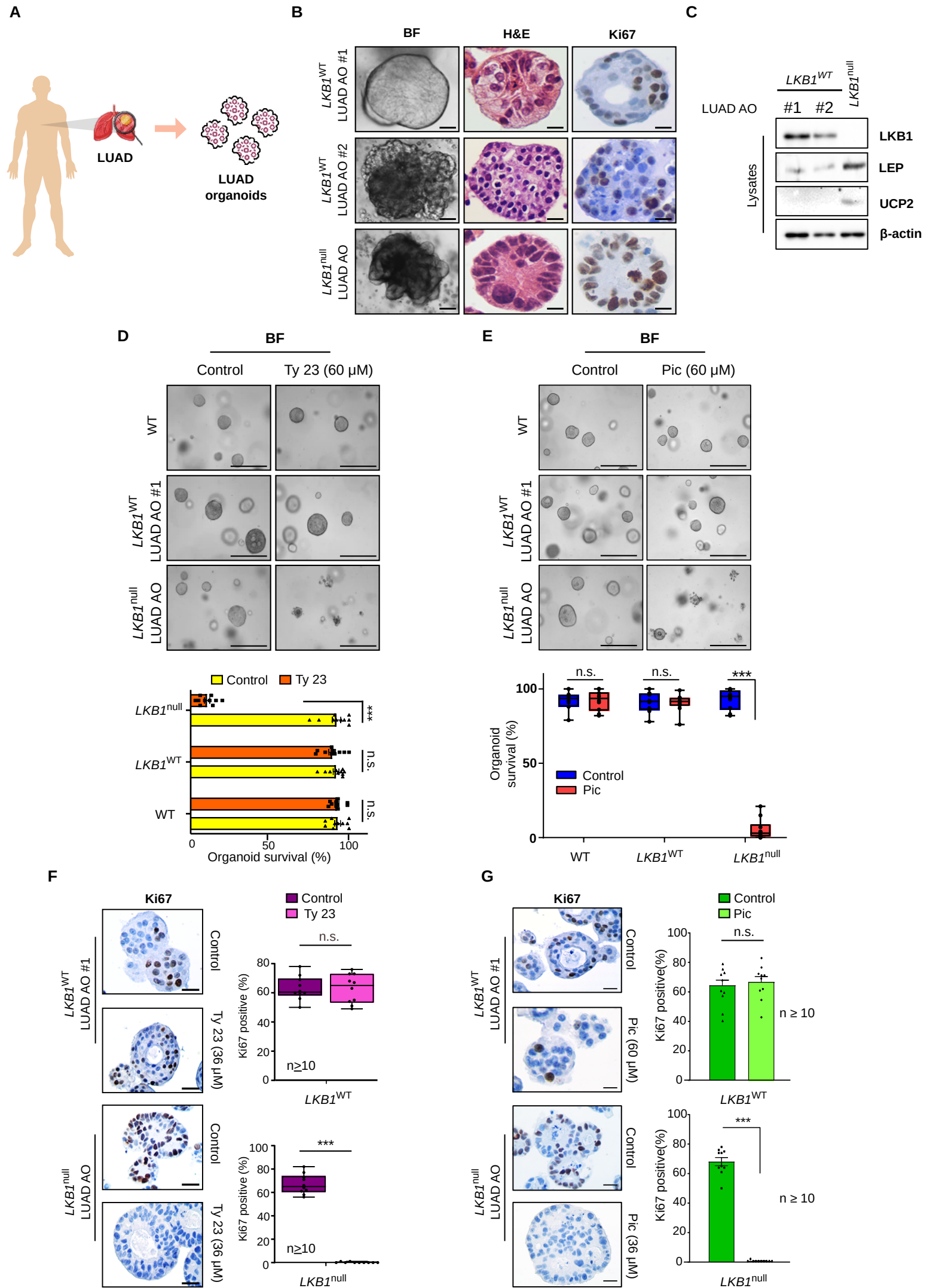

Supplement: Supplementary file 9 — Additional file 9: Supplementary Fig. 4. LEP is upregulated in TP53KO/LKB1KO and LKB1null LUAD organoids accompanied by enhanced sensitivity to identified metabolic activators. (A) LUAD AOs were generated after surgical resection of tumour tissue from LUAD patients and subsequently subjected to thorough genetic and histological characterisation. (B) Representative images of human LUAD organoids stained for haematoxylin and eosin (H&E) and Ki67. Two LKB1-proficient and one LKB1-null LUAD organoid lines were utilized (LKB1wt LUAD AO #1/#2 and LKB1null LUAD AO, respectively). Scale: 30 μm. (C) Western blotting from lysates of all LUAD AOs in (B) displaying upregulated LEP and UCP2 in LKB1null LUAD AOs. (D-E) Representative bright-field (BF) images and survival assessment of WT, LKB1wt or LKB1null LUAD organoid cultures receiving or not treatment with (D) tyrphostin 23 (60 µΜ) or (E) piceatannol for 5 days. Only LKB1null AOs die at the end of the treatment. Scale: 60 μm. (F-G) Immunohistochemistry on LKB1wt and LKB1null LUAD AOs to assess Ki67 levels upon treatment or not with (F) tyrphostin 23 or (G) piceatannol, at a less lethal dose (36 µΜ). LKB1null AOs display complete loss of proliferative capacity upon treatment. ***P < 0.001, of Student’s t-test. Error bars indicate s.e.m. N.s.; non-significant. Data shown are representative of at least 3 independent experiments. [file 12943_2024_2061_MOESM9_ESM.pdf]

Figure S5

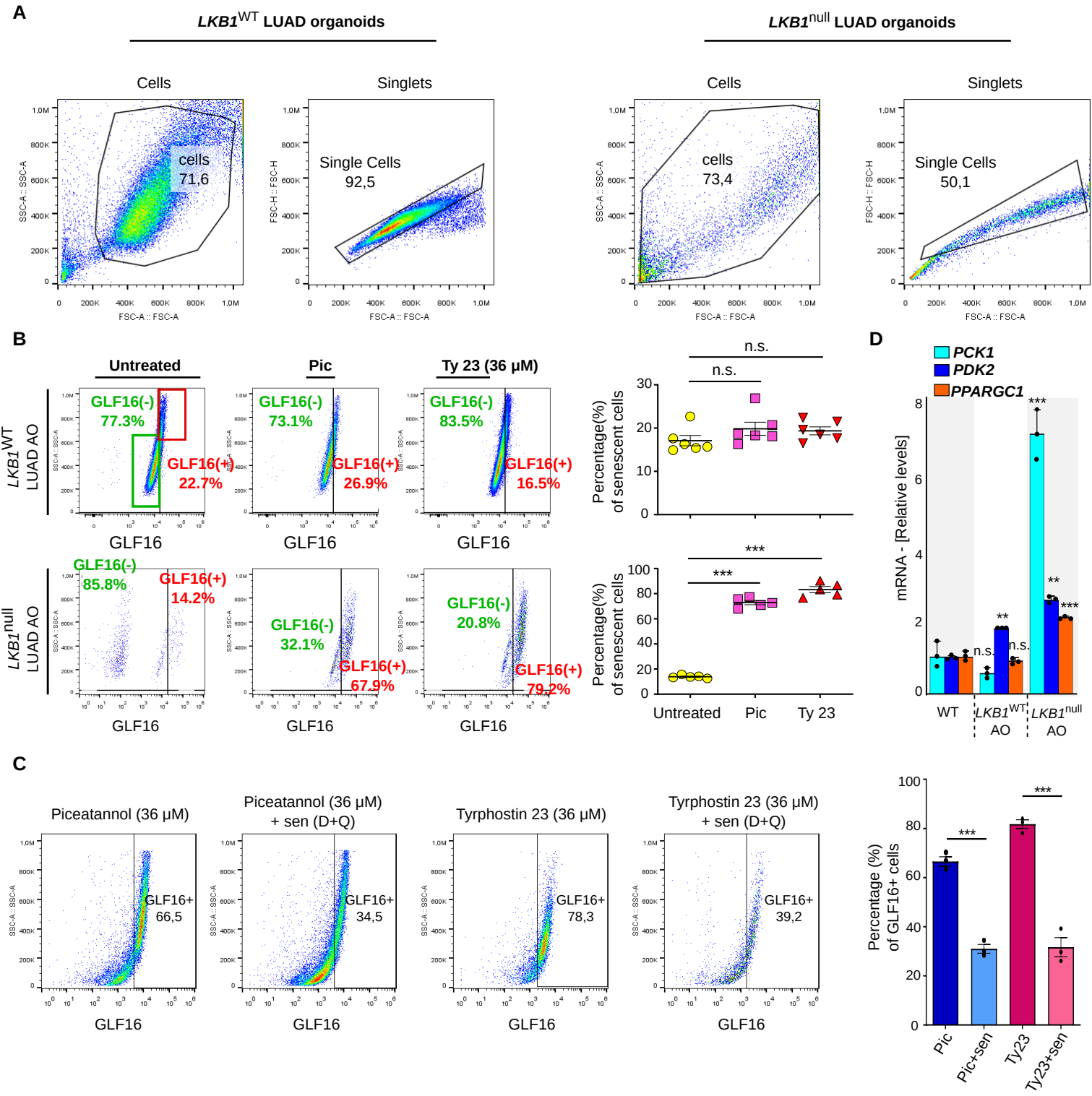

Supplement: Supplementary file 10 — Additional file 10: Supplementary Fig. 5. Treatment of LKB1null LUAD organoids with metabolic stressors induces senescence. (A) Gating parameters on LKB1wt and LKB1null samples for FACS experiments. (B) FACS plots and quantification of the percentage of senescent cells upon treatment of LKB1wt or LKB1null LUAD organoids with piceatannol or tyrphostin 23 at sublethal concentrations (36 µM) versus untreated counterparts. Senescence was assessed by implementing the rapid senescence detection fluorophore-conjugated GLF16 compound [10]. Treatment of organoids with either metabolic stressors led to significant induction of senescence compared to control only in LKB1-null conditions. (C) FACS plots and relevant quantification confirming that combined use of senolytics dasatinib and quercetin (D + Q) on LKB1null LUAD organoids receiving treatment with piceatannol or tyrphostin 23 significantly eradicates senescent cell populations. Piceatannol/tyrphostin 23-mediated growth arrest is achieved even at low concentrations, via senescence induction. (D) mRNA levels of gluconeogenesis genes in LKB1wt and LKB1null AOs. The gluconeogenesis regulator PDK2 is found dramatically increased in the absence of LKB1. ***P < 0.001 and **P < 0.01, of Student’s t-test. Error bars indicate s.e.m. N.s.; non-significant. Data shown are representative of at least 3 independent experiments. [file 12943_2024_2061_MOESM10_ESM.pdf]

Figure S6

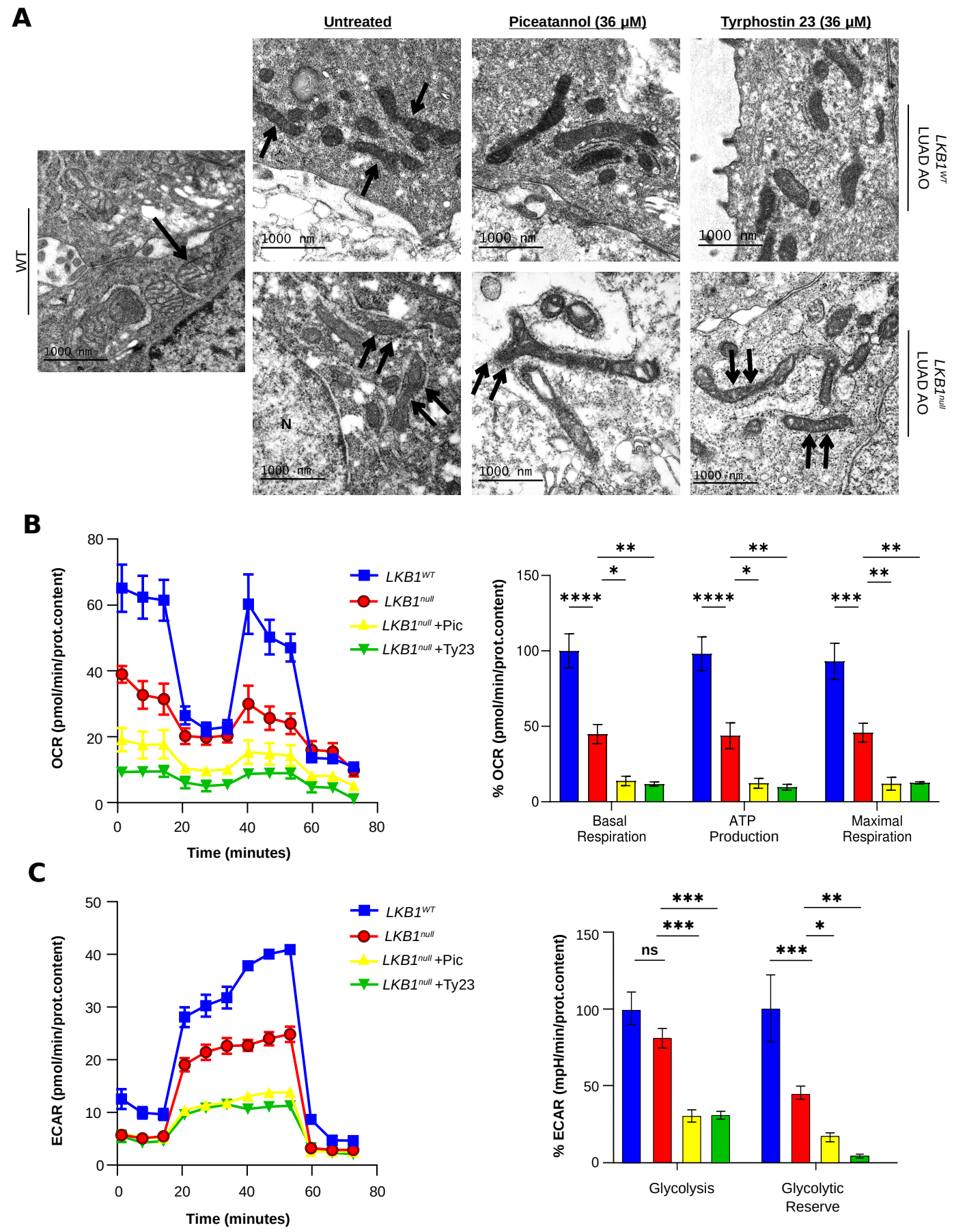

Supplement: Supplementary file 11 — Additional file 11: Supplementary Fig. 6. LKB1 deficiency is accompanied by a deregulated mitochondrial and bioenergetic profile exacerbated by piceatannol or tyrphostin 23 treatment. (A) Transmission electron microscopy (TEM) pictures of indicated organoid lines before and after treatment with piceatannol or tyrphostin 23 (36 µM). WT AOs were used as reference for normal mitochondrial structure, indicated with single black arrow. Most cells of LKB1wt LUAD AOs displayed normal mitochondria, with or without treatment. Most cells of LKB1null LUAD AOs exhibited aberrant mitochondrial structure, further deteriorated upon piceatannol or tyrphostin 23 treatment. Double black arrows indicate aberrant mitochondrial morphology, such as partial loss of cristae, cristae widening and mitochondrial elongation. N: nucleus; Scale bar: 1000 nm. (B) Oxygen Consumption Rate (OCR) of indicated untreated or piceatannol/tyrphostin 23-treated cells was determined using the Seahorse XFe96, via metabolic flux analysis. LKB1null LUAD cells display a decrease in both basal and maximal respiration, as well as mitochondrial ATP production, compared to LKB1wt LUAD counterparts, and the effect is exacerbated upon compound treatment. (C) Extracellular Acidification Rate (ECAR) of indicated untreated or piceatannol/tyrphostin 23-treated cells was determined as in (B). LKB1null LUAD cells show a decrease in glycolytic reserve, which is further reduced upon compound treatment. Data represent the % average ± SD over LKB1wt LUAD cells, n = 3. Two-way ANOVA, ns = not significant, *P < 0.05, **P < 0.005, ***P < 0.0005, ****P < 0.00005. [file 12943_2024_2061_MOESM11_ESM.pdf]

Figure S7

A

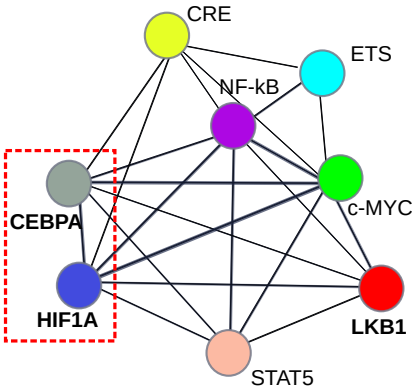

B

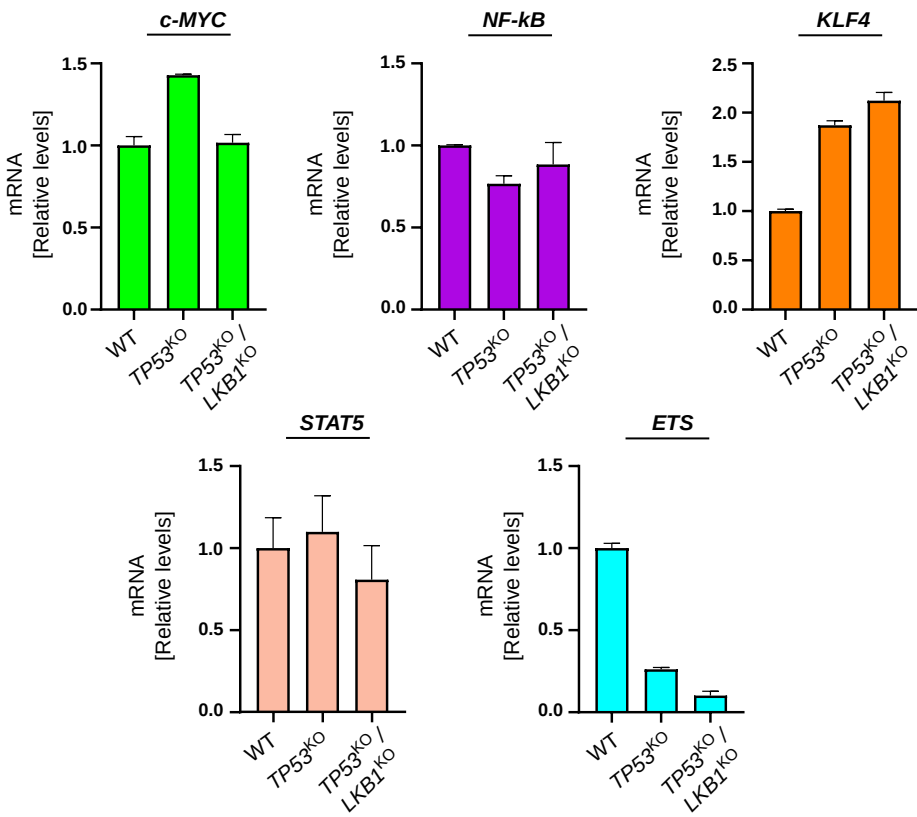

C

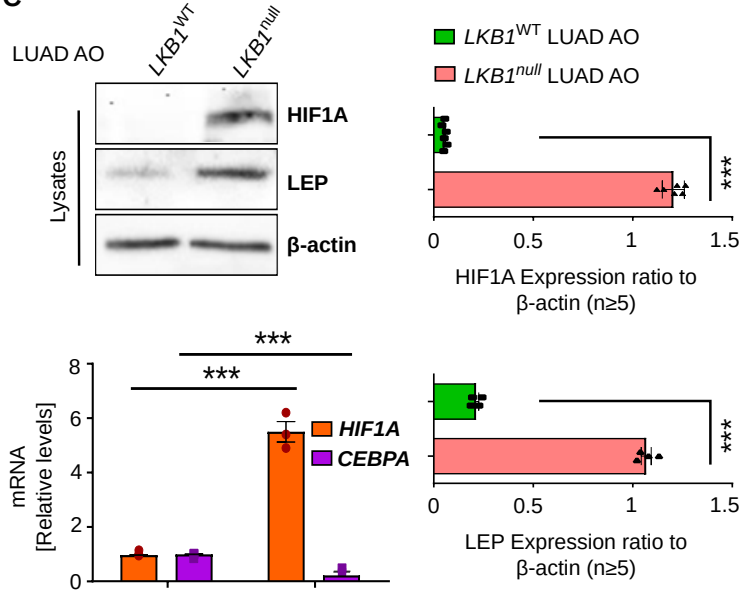

D

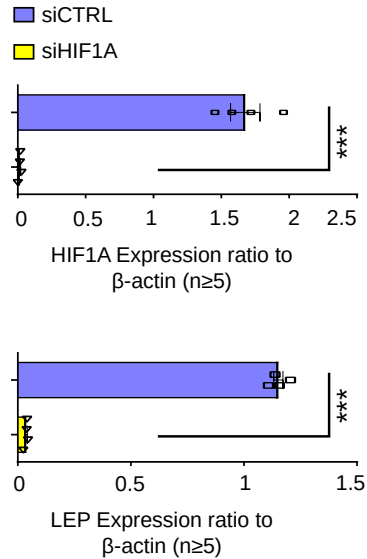

E

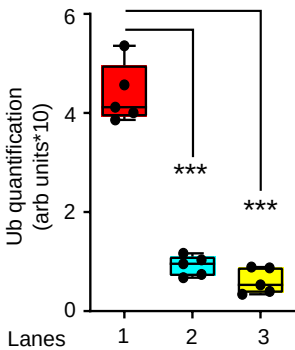

F

LUAD

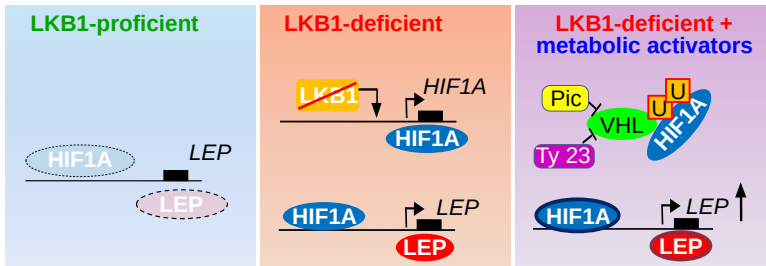

Supplement: Supplementary file 12 — Additional file 12: Supplementary Fig. 7. LKB1-dependent changes in transcription factor levels identified as potential regulators of the LEP gene. (A) STRING network representing the functional relationship between LKB1 and the potentially LEP-regulating transcription factors identified in Fig. 1M. (B) q-PCRs of indicated genes in CRISPR/Cas9-engineered AOs displaying either non-significant changes between conditions (WT, p53KO, p53KO/LKB1KO AOs) or expression changes likely not attributed to LKB1 alone. (C) Western blotting and q-PCR for indicated markers in LKB1wt and LKB1null LUAD AOs. Western blotting differences are verified among multiple independent experiments (n ≥ 5). (D) Densitometry for Western blotting in Fig. 1P. (E) Quantification of ubiquitination levels across different conditions in Fig. 2B. (F) Schematic depicting the identified LKB1-dependent regulation of LEP via HIF1A, in LKB1-proficient and -deficient LUADs. In the presence of LKB1, low expression of HIF1A results in lack of HIF1A-mediated activation of LEP transcription, with a considerable impact on overall LEP levels. In contrast, upon loss of LKB1, upregulation of HIF1A leads to high-affinity LEP promoter occupancy by HIF1A, resulting in significantly increased LEP levels. Additional treatment with piceatannol or tyrphostin 23 further stabilises HIF1A via inhibition of VHL-mediated HIF1A ubiquitination, resulting in hyperactivation of LEP. ***P < 0.001, of Student’s t-test. Error bars indicate s.e.m. N.s.; non-significant. Data shown are representative of at least 3 independent experiments. [file 12943_2024_2061_MOESM12_ESM.pdf]

Figure S8

A

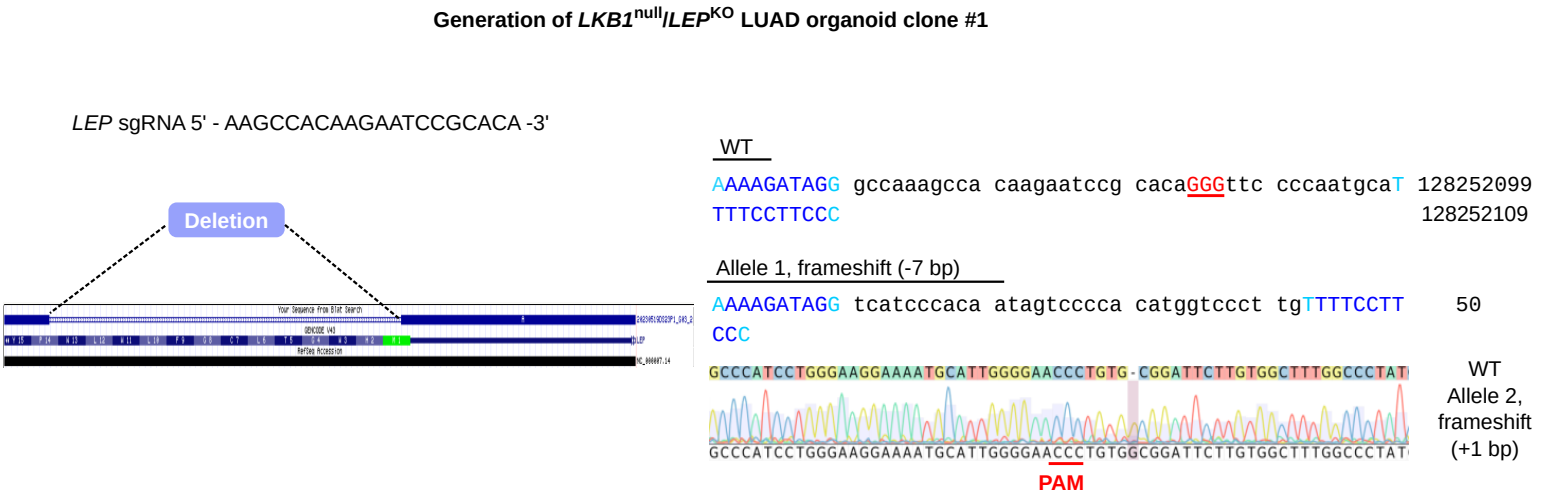

B

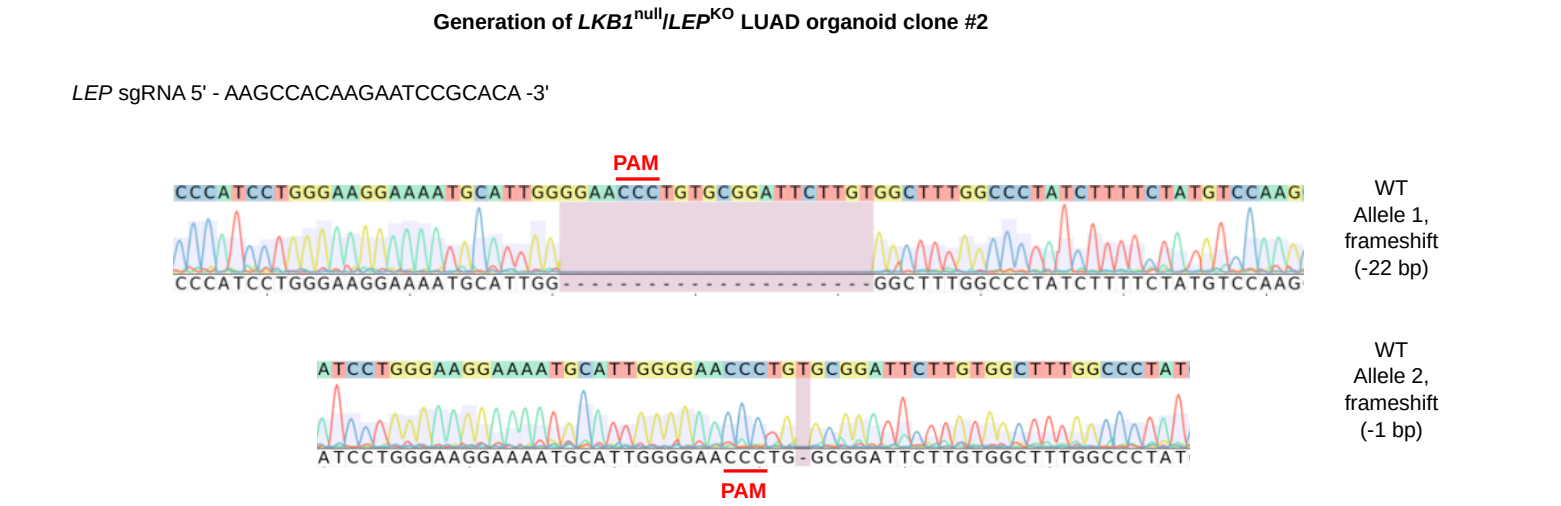

Supplement: Supplementary file 13 — Additional file 13: Supplementary Fig. 8. Sequencing results verifying successful LEP KO in LKB1null LUAD organoids. (A-B) PCR amplification products of the mutated alleles were obtained using primers flanking the targeted exons. PCR products were subjected to TA cloning into a pGEM-T vector and subsequent sequencing revealed indels at the expected locations. For each sgRNA used, the WT sequences and the targeted alleles are displayed. PAM sequences are underlined in red. In order to capture extensive genomic rearrangements the BLAT pairwise sequence alignment algorithm was used, whereas less extensive rearrangements are depicted using the sangerseq_viewer Python package. When the BLAT algorithm is used, nucleotides marked in blue are successfully aligned between WT and mutant alleles, whereas lower case nucleotides in black indicate the introduction of an indel. [file 12943_2024_2061_MOESM13_ESM.pdf]

Figure S9

A

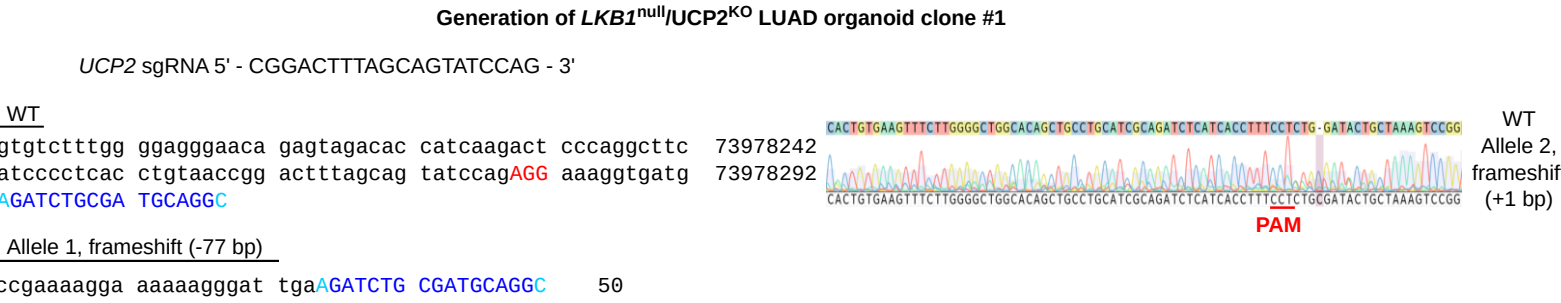

B

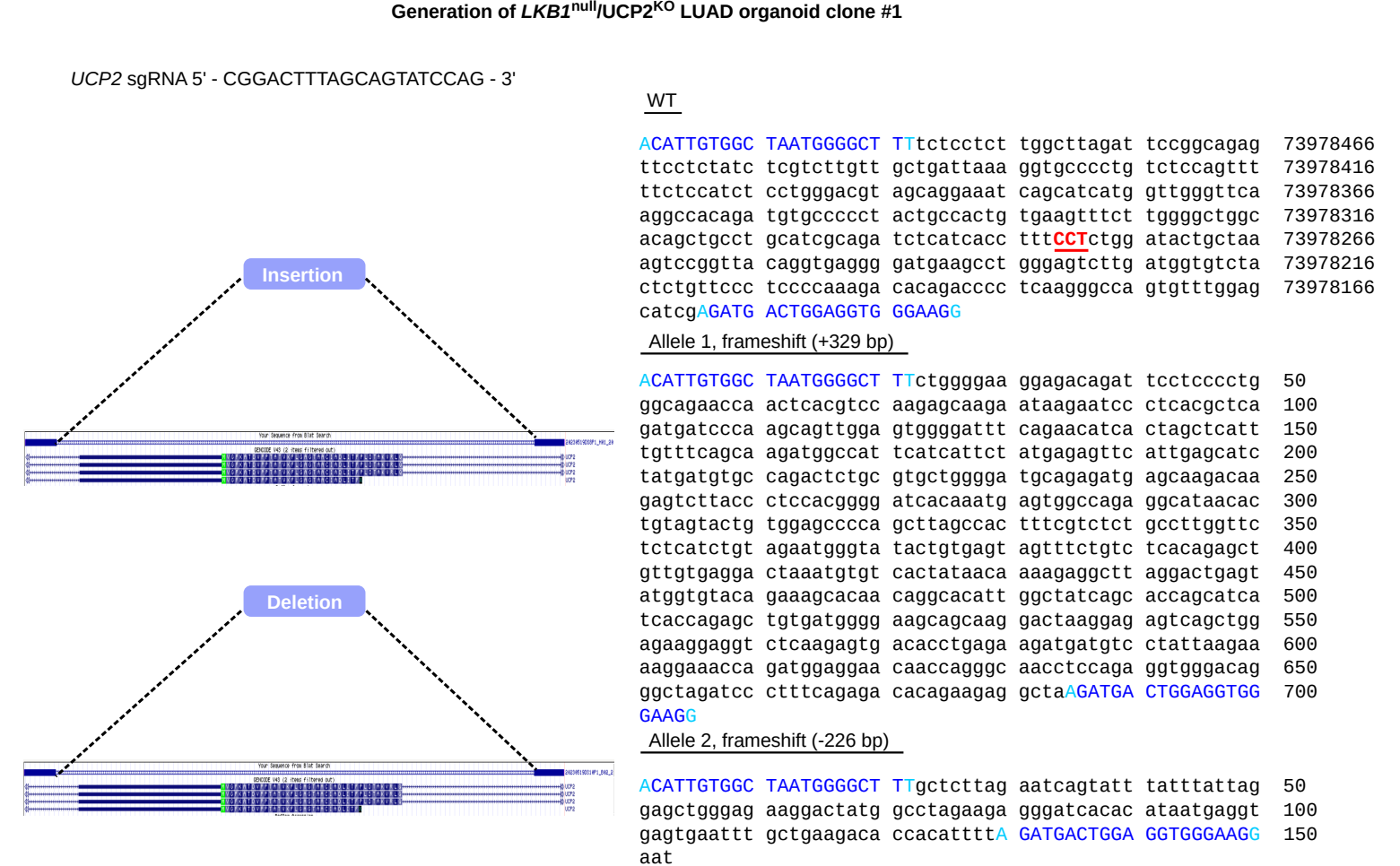

C

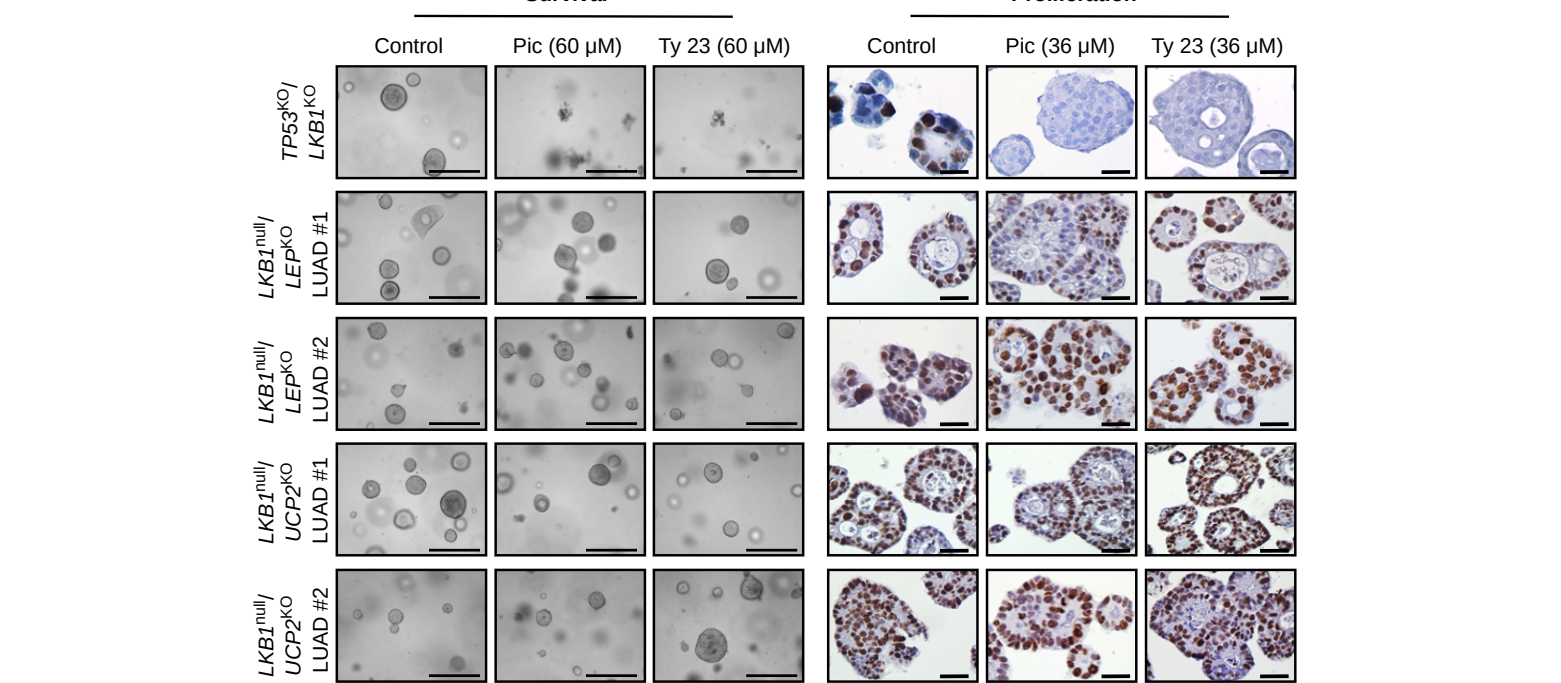

Supplement: Supplementary file 14 — Additional file 14: Supplementary Fig. 9. Sequencing results verifying successful UCP2 KO in LKB1null LUAD organoids. (A-B) PCR amplification products of the mutated alleles were obtained using primers flanking the targeted exons. PCR products were subjected to TA cloning into a pGEM-T vector and subsequent sequencing revealed indels at the expected locations. For each sgRNA used, the WT sequences and the targeted alleles are displayed. PAM sequences are underlined in red. In order to capture extensive genomic rearrangements the BLAT pairwise sequence alignment algorithm was used, whereas less extensive rearrangements are depicted using the sangerseq_viewer Python package. When the BLAT algorithm is used, nucleotides marked in blue are successfully aligned between WT and mutant alleles, whereas lower case nucleotides in black indicate the introduction of an indel. (C) Representative bright-field (BF) images and Ki67 stainings of the indicated AO lines receiving or not treatment with piceatannol or tyrphostin 23 for 5 days. See also Fig. 2G for respective quantification Scale: 30–60 μm. [file 12943_2024_2061_MOESM14_ESM.pdf]

Figure S10

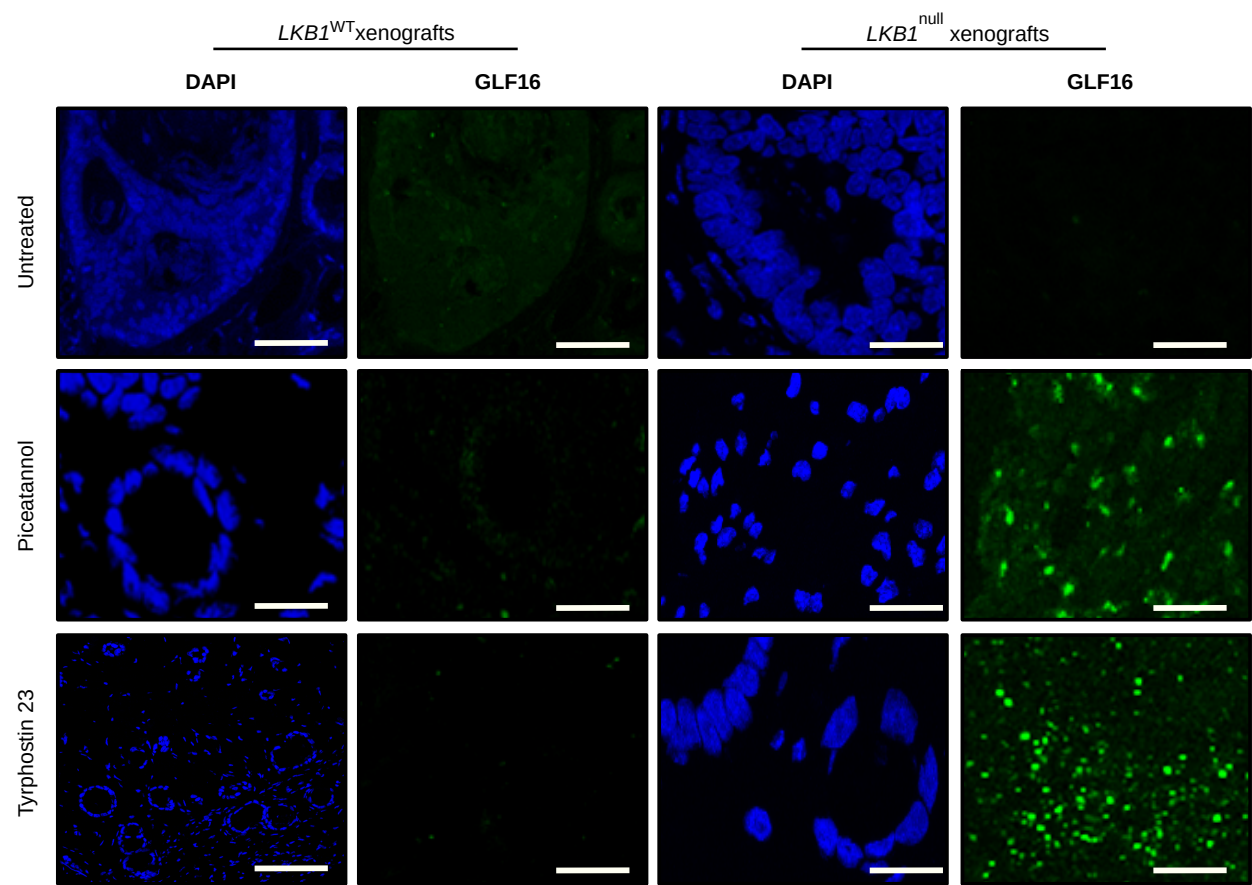

Supplement: Supplementary file 15 — Additional file 15: Supplementary Fig. 10. Piceatannol and Tyrphostin 23 exclusively suppress transplanted LKB1-deficient human LUAD organoids in vivo. GLF16 immunofluorescence in untreated, piceatannol- or tyrphostin 23-treated LKB1wt and LKB1null mouse xenografts. Treatment with either compound induces in vivo senescence only in LKB1null mouse xenografts. Scale: 30 μm. Quantification is presented in Fig. 2M. [file 12943_2024_2061_MOESM15_ESM.pdf]

Figure S11

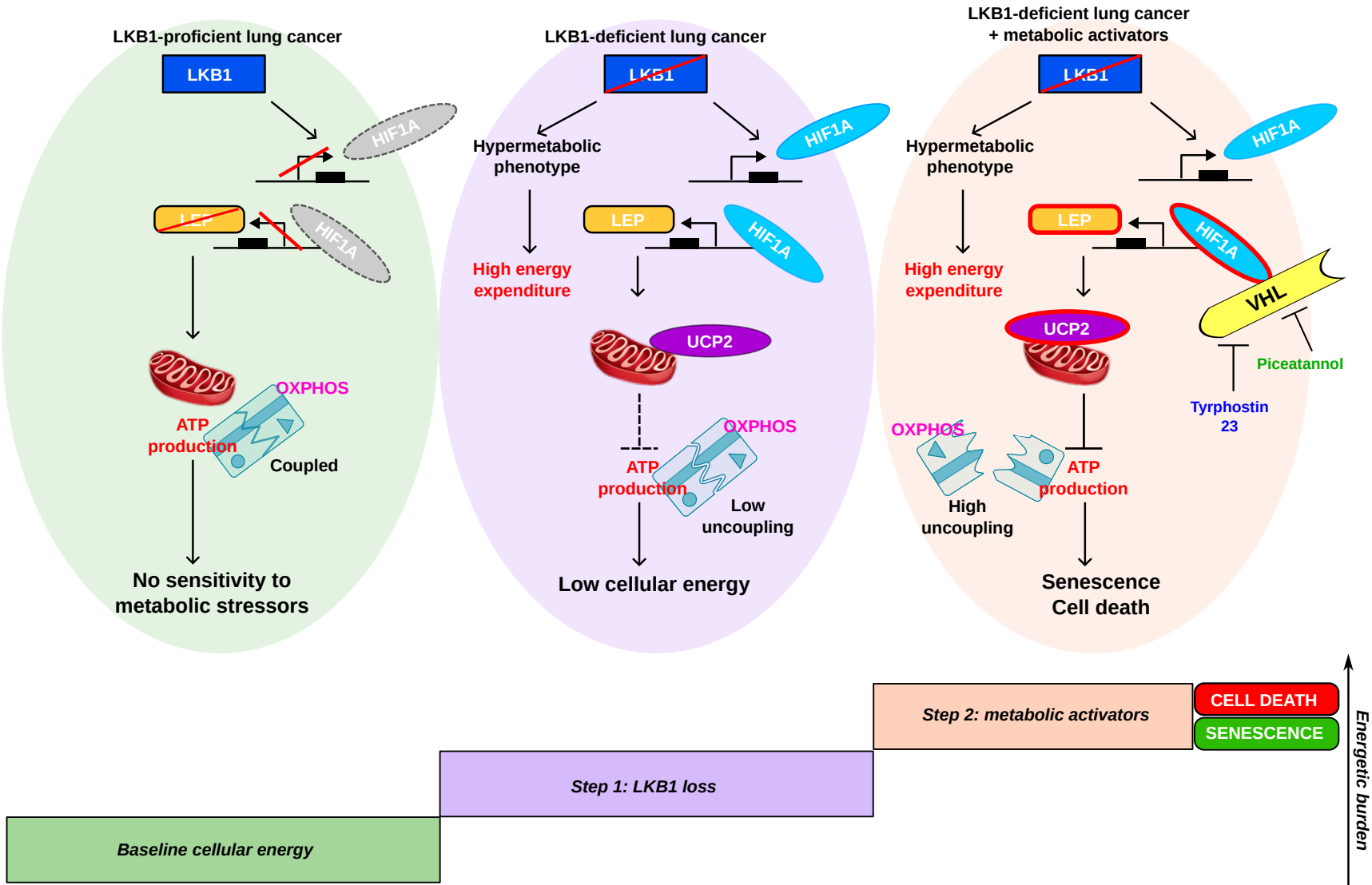

Supplement: Supplementary file 16 — Additional file 16: Supplementary Fig. 11. Model depicting a biphasic escalation of energetic stress in human LKB1-null LUAD and tumour elimination strategy. Upon loss of LKB1 in lung cancer, cells already enter a state of severe metabolic stress, characterised by a marked imbalance between glycolysis and gluconeogenesis and acquisition of a hypermetabolic phenotype leading to accelerated energy expenditure. However, LEP is additionally upregulated by LKB1 loss at the promoter level through HIF1A stabilisation, resulting in activation of a HIF1A-UCP2 signaling axis culminating in partial uncoupling of oxidative phosphorylation from ATP production, which reduces cellular energy reservoirs, contributing to metabolic stress. Additional treatment of LKB1-deficient cells with metabolic activators piceatannol and tyrphostin 23 further exacerbates the effects of the HIF1A-UCP2 axis, thus selectively conferring senescence-driven growth arrest or lethality to LKB1-deficient cells, via metabolic exhaustion above a sustainable threshold. In contrast, LKB1-proficient tumours fail to sufficiently upregulate the HIF1A-UCP2 axis, rendering tumour cells incapable of acquiring susceptibility to metabolic activators. [file 12943_2024_2061_MOESM16_ESM.pdf]
