# Supplementary material for: Loss of the tumour suppressor LKB1/STK11 uncovers a leptin-mediated sensitivity mechanism to mitochondrial uncouplers for targeted cancer therapy
Source: Mol Cancer. 2024 Jul 25;23:147. doi: 10.1186/s12943-024-02061-4 (PMC11270803; doi:10.1186/s12943-024-02061-4)
Supplement: Supplementary file 17 — Additional file 17 [file 12943_2024_2061_MOESM17_ESM.docx]

**Materials and Methods**

**Zebrafish strains and Screening Methods**

Zebrafish were handled in compliance with the local animal welfare regulations and were maintained according to standard protocols ([zfin.org](http://zfin.org)). Their culture was approved by the local animal welfare committee (DEC) of the University of Leiden and all protocols adhered to the international guidelines specified by the EU Animal Protection Directive 2010/63/EU. Genotype analysis for *lkb1*-mutant embryos was performed with SNP-specific PCR analysis.

**RNA extraction for RT-qPCR, glucose metabolism array and transcriptome analysis in zebrafish**

Trunks of embryos from single-matings were individually stored in RNAlater (Invitrogen, AM7021) until genotyped. Total RNA was isolated from 10 trunks of either *lkb1*, het or wt trunks, with the RNaqueous micro Total RNA isolation kit (Thermo Fisher Scientific, AM-1931) following manual. Briefly, 10 trunks were lysed in 200 μL Lysis solution, supplemented with 100 μL 100% EtOH and applied to the column. Total mRNA was eluted in 2x10 μL Elution solution. Concentrations were measured using Nanodrop (ND-1000).

**Zebrafish gene expression analysis**

500-1000 ng of total RNA was reverse transcribed using iScript cDNA synthesis kit (BioRad, 1708890). 1:10 Diluted cDNA was analyzed with real-time quantitative RT-PCR performed in a Bio-Rad CFX96 system. For the glucose metabolism array (RT² Profiler™ PCR Array Zebrafish Glucose Metabolism (SABiosciences, PAZF-006Z)), plates with aliquoted primers for the indicated genes were used. Data analysis of qPCR results represents data from three independent experiments. Error bars represent the means ± standard errors of the means (SEM). Statistical significance was calculated using 2way ANOVA using a Tukey’s multiple comparisons test.

**Transcriptome analysis**

Total RNA isolated from trunks of 5, 7 dpf wt and *lkb1* larvae, and 11 dpf wt larvae were analyzed in triplicates using an Illumina sequencing pipeline. The 51-bp single-end reads were mapped to the *Danio rerio* genome (Zv9) using TopHat (2.0.12) [1]. Statistical analysis was performed using the online platform SeqMonk (Babraham Bioinformatics). The differential expression of genes was determined using the build-in scripts for DESeq2 [2] and EdgeR [3]. A False Discovery Rate (FDR) for differential expression lower than 0.05, P-values lower than 0.05 and a log2 fold-change more then 1 were considered significant.

**Synthetic lethality screen**

Larvae were treated with compounds of the ENZO Kinase inhibitors (ENZO life sciences, BML-2832, 80 inhibitors) and phosphatase inhibitors (ENZO, BML-2834, 30 inhibitors) between 4 and 7 dpf. Stocks were diluted in embryo medium for treatment with a final concentration of 10 μM, 0.2% DMSO. Screening was performed in 96-well plates with 3 larvae per well, using 8 wells per compound. Larvae that died during treatment and all surviving larvae at 8 dpf were genotyped for the *lkb1* gene. Compounds that selectively killed *lkb1* larvae were validated in a secondary screen in 6-well plates using 24 larvae per treatment. The secondary screen and validations were performed in triplicate.

**Compound treatments**

Wild type or *lkb1*-mutant zebrafish embryos were treated from 4 to 9 dpf in embryo-medium at 28 °C with either of the following treatments: 25-50 μM Piceatannol (ENZO life sciences, ALX-270-202), 25 μM Tyrphostin 23 (ENZO life sciences, BML-EI191), or 0.2% DMSO. Stock solutions were prepared in DMSO and diluted in embryo medium for treatment with a final concentration of 0.2% DMSO. Larvae were collected at the specified time points and genotyped for the *lkb1* gene. Each treatment was performed in triplicate with 48 embryos per experiment. Kaplan-Meier curves were derived with Graphpad Prism 7c, combining all three experiments. Treatment curves were compared to control curves using the Log-rank (Mantel-Cox) test, with P-value <0.05 as statistically significant.

**Airway organoid cultures**

Airway organoid cultures were established from normal or LUAD patient samples as previously described [4]. Briefly, lung cell pellets were resuspended in 10 mg/mL cold Cultrex growth factor reduced BME type 2 (Trevigen-3533-010-02) and 40 μL drops of BME-cell suspension were allowed to solidify on prewarmed 24-well suspension culture plates (Greiner- M9312) at 37 °C for 30 min. Upon completed gelation, 1 mL of lung organoid medium was added to each well and plates transferred to humidified 37°C / 5% CO_2_ incubators at ambient O_2_. Medium was changed every 3 days and organoids were passaged approximately once a week by both enzymatic dissociation in 2 ml TrypLE Express (Invitrogen-12605036), incubation for 15 min at 37°C, and mechanical shearing through flamed glass Pasteur pipettes. Following the addition of 10 mL AdDF+++ (Advanced DMEM/F12 containing 1× Glutamax, 10 mM HEPES, and penicillin/streptomycin) and centrifugation at 500 x g respectively, single cells and organoid fragments were resuspended in cold BME and reseeded as above at various ratios allowing for the formation of new organoids. The collection of tissues for the generation of airway organoids was carried out according to the European Network of Research Ethics Committees (EUREC) guidelines and the local NKUA Medical School Bioethics committee (Protocol number: 571). All organoid lines were generated from patients who signed informed consent forms and their personal information remained anonymous.

**CRISPR/Cas9-mediated airway organoid engineering and sequencing**

The organoid lipofection protocol was followed as previously described [5, 6] with a few adaptations. Briefly, human airway organoids grown in full organoid media were trypsinized for 20 min at 37^o^C and sheared with a glass pipette to produce single cells. After dissociation, cells were resuspended in 450 μL Advanced DMEM/F-12 (Thermo Scientific) and plated in 48-well plates at a confluency of >90%. For each transfection, a total of 1.5 μg of plasmid DNA in 50 μL of serum-free medium (Opti-MEM; Gibco) were mixed with 4 μL of Lipofectamine 2000 (Invitrogen) diluted in 50 μL of serum-free medium making up a total volume of 100 μL, which was then added to the cells. The plate was centrifuged at 600 x g at 32 ^o^C for 1 h and incubated for 4-5 h at 37^o^C before the cells were embedded in Basement Membrane Extract (BME) in full organoid medium. 10 μM of Nutlin-3 (Cayman Chemical) were added to the cultures 1 day after transfection and maintained for 16-21 days for mutant *TP53* selection. Puromycin (Gibco; 1:5000 concentration) selection was applied for *LKB1, LEP* and *UCP2* mutants 1 day after transfection and maintained for 4 days. For clonal expansion, single organoids were picked. The sgRNA *TP53* sequence was specifically designed to target exon 3 of the *P53* gene and was: 5’-GGATGATTTGATGCTGTCCC-3’; the sgRNA *LKB1* sequence was specifically designed to target exon 1 of the *LKB1* gene and was: 5’-AGCTTGGCCCGCTTGCGGCG-3’; the sgRNA *LEP* sequence was specifically designed to target exon 1 of the *LEP* gene and was: 5’- AAGCCACAAGAATCCGCACA-3’ and the sgRNA *UCP2* sequence was specifically designed to target exon 1 of the *UCP2* gene and was: 5’-CGGACTTTAGCAGTATCCAG-3’. For sequencing, genomic DNA was isolated using Viagen Direct PCR (Viagen). Primers for PCR amplification using GoTaq Flexi DNA polymerase (Promega) were: *P53*_for, 5’-CAGGAAGCCAAAGGGTGAAGA-3’, *P53*_rev, 5’-CCCATCTACAGTCCCCCTTG-3’; *LKB1*_for, 5’-AGGGAAGTCGGAACACAAGG-3’, *LKB1*_rev, 5’-GACCCCAGCAAGCCATACTTA-3’; *LEP*_for, 5’- ATCCCCGTCTGGTAATGTGG-3’, *LEP*_rev, 5’- TGGCATTCAGGAGGCGTTC-3’; *UCP2*_for, 5’- ACATTGTGGCTAATGGGGCTT-3’, *UCP2*_rev, 5’- CCTTCCCACCTCCAGTCATC-3’. PCR products were purified using the QIAquick PCR purification kit (Qiagen) and cloned into pGEM-T Easy vector system I (Promega), followed by sequencing using the T7 primer. The plasmid vector used in transfections was obtained from Addgene (#62988) [7].

**RNAi transfection in airway organoids**

Organoid dissociation to single cells was performed as described in the previous section. The cell pellet was resuspended in 1.5 mL of Advanced DMEM (in the absence of Penicillin/Streptomycin) and placed in a well of a 6-well plate at ~90% confluency, where 0.5 mL of siRNA/lipofectamine mix was added according to the manufacturer’s instructions. For each transfection, 100 nM of siRNA and 5μL lipofectamine 2000 (Invitrogen) were used, diluted in Opti-MEM medium (Gibco). The plate was then centrifuged at 600 x g for 1h at 32^o^C and incubated for 5h at 37^o^C before the cells were seeded in Basement Membrane Extract (BME) in full organoid medium. The organoids were harvested 48h after transfection for qPCR and Western blot analysis. A SMARTpool siRNA targeting HIF1A (FlexiTube GeneSolution GS3091, Cat No 1027416) was used.

**Chromatin immunoprecipitation (ChIP)**

Chromatin preparation and Immunoprecipitation was performed as previously described [8-10] with modifications. Airway organoids were harvested using ice-cold Cell Recovery Solution for 45 min at 4 ^o^C. After washing with PBS to remove residual BME, organoids were homogenized in PBS using a Dounce glass grinder. Samples were cross-linked with 1% formaldehyde (A0877,0250; AppliChem) incubated for 10 min at Room Temperature, followed by quenching with 0.125 M glycine at Room Temperature for 7 min and centrifugation (4,000 rpm, 7 min, 4°C). Pellets were washed with PBS, resuspended in lysis buffer (50 mM Hepes, pH=7.9, 140 mM NaCl, 1 mM EDTA, 10% glycerol, 0.5% NP-40, and 0.25% Triton X-100 supplemented with Protease Inhibitors-PI, Roche, 5892970001) and incubated for 20 min at 4°C. Lysates were centrifuged (4,000 rpm, 10 min, 4°C) and pellets were washed twice with wash buffer (10 mM Tris-HCl, pH=8.1, 200 mM NaCl, 1 mM EDTA, pH 8, and 0.5 mM EGTA, pH 8). For the shearing step, Sonication buffer (0.1% SDS, 1 mM EDTA, and 10 mM Tris pH=8.1, supplemented with PI) was added, and chromatin shearing was performed in the Covaris S2 sonicator (Covaris) using milliTUBE AFA Fiber 1 mL tubes (Covaris, 520130) for 10 min (duty factor, 75; peak power, 25; cycles per burst, 200) allowing the shearing of chromatin within a range of 250–500 bp DNA fragments. Samples were centrifuged (13,000 rpm, 30 min, 4°C), and the supernatants were collected. Triton X-100 and NaCl to final concentrations of 1% and 150 mM, respectively, were added in the samples. For the pre-clearing step, Protein G-Dynabeads (10003D; Invitrogen) were equilibrated in immunoprecipitation buffer (0.1% SDS, 1 mM EDTA, 10 mM Tris, pH 8.1, 1% Triton X-100, and 150 mM NaCl), using 50 μL from Protein G-Dynabeads per 75 μg of chromatin.

Immunoprecipitation was performed by incubating 75 μg of chromatin with 10 μg of anti-HIF1α (ab179483, Abcam) , 10 μg anti-CEBPA (sc-365318, Santa Cruz) or 10 μg of normal rabbit IgG antibody (Cell Signaling Technology, 2729s), rotated in an orbital shaker overnight at 4°C. 50 μL of Protein G-Dynabeads (10003D; Invitrogen) per 10 μg of antibody were equilibrated in immunoprecipitation buffer and then incubated with the chromatin-antibody solution at 4°C for 4 hours. The recovered resin was washed twice with immunoprecipitation buffer, once with a wash buffer consisting of 50 mM Tris-HCl pH 8, 150 mM NaCl, 2 mM EDTA, 1% NP-40, 0.5% Na-deoxycholate, 0.1% SDS and 250 mM LiCl and once with TE buffer (10 mM Tris-HCl pH=8, 10 mM EDTA). The captured chromatin fragments were eluted with addition of Elution Buffer (0.5% SDS, 20 mM Hepes, pH=7.9, and 1 mM EDTA) and incubation at 65°C for 30 min with frequent vortexing. Chromatin samples were subjected to reverse cross-linking by incubating with 20 mg RNase A (R6513, Sigma-Aldrich) at 37°C for 30 min and 30 mg proteinase K (031158360, Roche) at 50°C for 30 min, followed by the addition of 0.6 M NaCl and overnight incubation at 65°C. All DNA present in each sample was purified with NEB beads (744970.50, Macherey-Nagel) and eluted in TE buffer.

ChIP q-PCR data were analyzed by measuring the amount of immunoprecipitated DNA relative to input DNA and normalized to IgG. ChIP qPCR primer sequences targeting the promoter and/or proximal regulatory regions are listed below: *HIF1A*_for, 5’-TTCTCGCTCCTACCAGCCACC-3’, *HIF1A*_rev, 5’-TAGCCCTGGGCCCGCAG-3’; *CEBPA*_for, 5’- GGAGCTGGCGCTAGAAATGC-3’, *CEBPA*_rev, 5’- TTCCTACGGGGCTCCATGC-3’.

***In vivo* ubiquitination assay**

*LKB1^null^* LUAD AOs were treated or not with 36-60 μΜ of either piceatannol or tyrphostin 23. 48 h following drug treatment, cells were additionally treated with MG‐132 proteasome inhibitor (C2211, Sigma‐Aldrich) for 3 h at 30 µM and subsequently lysed in RIPA buffer (Tris–HCl at pH 8.0, 50 mM, NaCl 150 mM, SDS 0.1%, sodium deoxycholate 1%, Triton X‐100 1% and protease (#58440, Pierce, Thermo Scientific), phosphatase (#78420, Pierce, Thermo Scientific) inhibitor cocktail and N‐Ethylmaleimide deubiquitinase inhibitor (04259, Sigma‐Aldrich)). Protein lysates were precleared with protein G agarose beads (#16‐266, Millipore) for 1 h and then incubated with G‐protein beads bound to HIF1A antibody (ab179483) for 2 h at 4°C. Cell lysates and immunoprecipitates were analyzed in Western blotting.

**Immunoblotting**

To harvest organoids from culture, the culture medium was removed and organoids were placed in ice-cold Cell Recovery Solution for 45 min at 4 ^o^C. After spinning down at 500 x g for 5 min and discarding supernatant, the organoid pellet was washed with ice-cold PBS to remove residual BME. Total cell lysis and chemiluminescent signal detection were carried out as previously described [11-13]. Primary antibodies were used at the following concentrations: LKB1 (dilution 1:500, Santa Cruz; sc-374334), p53 (dilution 1:500, Santa Cruz; sc-126), HIF1A (dilution 1:1000, ab179483), Ub (dilution 1:1000, Cell Signaling; 3936), LEP (dilution 1:500, Abcam; ab16227), UCP2 (dilution 1:300, Santa Cruz; sc-390189), β-Actin (dilution 1:1000, Cell Signaling #4967). The HRP-conjugated anti-mouse (Cell Signaling #7076) and anti-rabbit (Cell Signaling #7074) secondary antibodies were used at a concentration of 1:1000.

**q-PCR**

Organoids were harvested as described in the Immunoblotting section above. RNA extraction, cDNA preparation and Quantitative Real-Time PCR were performed as previously described [11, 14]. The results were averaged from at least three independent experiments and further analyzed using the 2^-ΔΔCt method. The following primer sequences were used: *LEP*_for, 5’-GCTGTGCCCATCCAAAAAGTCC-3’ and *LEP*_rev, 5’- CCCAGGAATGAAGTCCAAACCG-3’; *PCK1*_for, 5’-CATTGCCTGGATGAAGTTTGACG-3’ and *PCK1*_rev, 5’-GGGTTGGTCTTCACTGAAGTCC-3’; *PDK2_*for, 5’-CGCTGGCTGGCTTTGGTTATG-3’ and *PDK2_*rev, 5’-ACAGGGCCTTGAGATAGATG-3’, *PPARGC1A*_for, 5’-CCAAAGGATGCGCTCTCGTTCA-3’ and *PPARGC1A*_rev, 5’-CGGTGTCTGTAGTGGCTTGACT-3’; *CREM*_for, 5’-CTCCTCCACCAGGTGCTACAAT-3’ and *CREM*_rev, 5’-AGCTCGGATCTGGTAAGTTGGC-3’, *IL6*_for, 5’-AGACAGCCACTCACCTCTTCAG-3’ and *IL6*_rev, 5’-TTCTGCCAGTGCCTCTTTGCTG-3’, *IL1B*_for, 5’-CCACAGACCTTCCAGGAGAATG-3’ and *IL1B*_rev, 5’-GTGCAGTTCAGTGATCGTACAGG-3’, C-*MYC*_for, 5’CCTGGTGCTCCATGAGGAGAC-3’ and C-*MYC*_rev, 5’-CAGACTCTGACCTTTTGCCAGG-3’; *NFkB*_for, 5’TGAACCGAAACTCTGGCAGCTG-3’ and *NFkB*_rev, 5’-CATCAGCTTGCGAAAAGGAGCC-3’; *KLF4*_for, 5’-CATCTCAAGGCACACCTGCGAA-3’ and *KLF4*_rev, 5’-TCGGTCGCATTTTTGGCACTGG-3’; *STAT5A*_for, 5’-AATGAGAACACCCGCAACG-3’ and *STAT5A*_rev, 5’- TTCCTGAAGTGGGCACTGA-3’; *ETS*_for, 5’GGGTGACGACTTCTTGTTTG-3’ and *ETS1*_rev, 5’-GTTAATGGAGTCAACCCAGC-3’; *CEBPA*_for, 5’CGGACTTGGTGCGTCTAAG-3’ and *CEBPA*_rev, 5’-GAGGCAGGAAACCTCCAAAT-3’; *HIF1A*_for, 5’-TATGAGCCAGAAGAACTTTTAGGC-3’ and *HIF1A*_rev, 5’-CACCTCTTTTGGCAAGCATCCTG-3’; *18S*_for, 5’-AGTCCCTGCCCTTTGTACACA-3’ and *18S*_rev, 5’- GATCCGAGGGCCTCACTAAAC-3’.

**Immunohistochemistry and immunofluorescence**

For immunohistochemistry, organoids were harvested as described in the Immunoblotting section above. Then, they were fixed in 4% paraformaldehyde for 20 min at room temperature, dehydrated and embedded in paraffin. 4 μm sections of the paraffin-embedded organoids and formalin-fixed paraffin embedded (FFPE) mouse tumours were used for histological analysis and immunohistochemistry. The sections were deparaffinized and hydrated. Antigen retrieval was performed by heating in 10 mM citric acid (pH 6.0) for 20 min and endogenous peroxidase was blocked with 3% H_2_O_2_ for 18 min in the dark. Blocking of non-specific binding sites was performed by using normal goat serum for 1h at room temperature (dilution 1:40; ab138478). Overnight incubation was performed at 4^o^C with the following primary antibodies: anti-Ki67 (dilution 1:200; ab16667), anti-TTF-1 (8G7G3; Dako) and anti-Cytokeratin (CAM 5.2) (BD Biosciences #345779). Positive signal was detected using the Dako REAL EnVision Detection System (Cat.no: K5007) by following the manufacturer’s instructions. Histological observations were performed on a ZEISS Axiolab5 microscope.

Immunofluorescence was carried out as previously described [15, 16]. Briefly, FFPE tissue sections (4 mm) were deparaffinized and rehydrated. The sections were immersed in citrate buffer (pH = 6) and antigenic epitope retrieval was performed in a steamer for 15 min. Non-specific epitope blocking was performed using sheep serum (S22, Merck Millipore). GLF16 was applied for 10 min (100 mg/mL) avoiding light exposure. The sections were washed 3 times for 10 min each, with the GLF16 diluent. Tissue autofluorescence was minimized using the TrueVIEW Autofluorescence Quenching Kit (Vector Laboratories Inc., CA, USA). The samples were counterstained with DAPI, mounted and visualized using the Leica TCS-SP8 confocal microscope at 20x, 40x and 63x objectives.

**Drug screening and Cell viability assay**

To prepare organoids for drug screening, dispase (D4693; Sigma-Aldrich) was added into organoid cultures grown in full organoid medium, at a final concentration of 2 U/mL, followed by incubation at 37 ^o^C for 1.5 h. Then, the organoids were collected, washed with DMEM and filtered through 70 μm cell strainers (#15-1070; Biologix) to exclude extremely large organoids. The number of organoids was counted using the Disposable Haemocytometer (C-Chip) and 1000 organoids per condition (at least 3 replicates for each condition) were seeded into BME in 6-well plates (Corning). After BME solidification, piceatannol and tyrphostin 23 were added into full organoid medium to achieve the following concentrations: 100 μΜ, 60 μΜ, 36 μΜ, 22 μΜ, 13 μΜ, 5 μΜ and 0 μΜ. 5 days post treatment, organoids from each well were resuspended in 120 μl full organoid medium divided over 3 wells of a 96-well plates, where the CellTiter-Fluor™ Cell Viability Assay reagent (Promega #G6081) was added, according to the manufacturer’s instructions. Readings were performed on a Varioskan LUX multimode microplate reader (Thermo Scientific).

**Detection of senescence by FACS**

Organoids were incubated with dispase (final concentration: 2 U/mL) for 1.5 h at 37°C, washed with DMEM and filtered through 70 μm cell strainers, as described in the previous section. Then, the organoids were counted and ~150.000 organoids per condition were seeded into BME in 6-well suspension plates (Corning). BME was allowed to solidify by incubating for 1 h at 37°C, and piceatannol or tyrphostin 23 were added in full organoid medium at a concentration of 36 μΜ for 5 days. To assess the effect of senolytics in combination with metabolic activators, Dasatinib (cat. No.6793, Tocris Bioscience, UK) and Quercetin (cat. No. 1592409, Sigma, USA) were additionally added 3 days after the initial treatment at a concentration of 15 μM each and were kept on for 2 days.

At the end of the treatment period, organoids were incubated with TrypLE Express for 20-30 min at 37°C and mechanically sheared using glass Pasteur pipettes to acquire single cells. The pellet was washed with PBS and fixed with 4% PFA for 15 min at room temperature. The cells were centrifuged at 500 x g for 5 min and PFA was removed. Permeabilization was achieved by adding 90% ice-cold methanol drop-wise in pre-chilled cells while vortexing. The cells were incubated for at least 10 min on ice and stored at -20°C until they were analyzed by FACS. Right before FACS, the samples were washed twice with PBS at 340 x g for 5 min at room temperature. Each sample was stained with 200 μL of diluted GLF16 (1:20 in PBS/2.5% DMSO/2.5% Tween-20) for 10 min at room temperature. 1 mL of GLF16 diluent (PBS/2.5% DMSO/2.5% Tween-20) was added on top of the compound. The samples were spun down at 340 x g for 5 min at room temperature and washed again with GLF16 diluent to ensure GLF16 removal. The pellet was then resuspended in 400 μL PBS/5% FBS and analyzed in a flow cytometer (Attune Nxt Flow Cytometer, Thermo Fisher Scientific Inc). Organoid cell populations were first visualized on a forward scatter (FS)/side scatter (SS) plot in order to exclude debris. Single cells were subsequently identified on a FSC-A/FSC-H scatter plot and gated. Senescent populations were evaluated using a SSC-A/APC-CY7-A plot based on unstained negative control cells.

**Organoid preparation for transmission electron microscopy (TEM)**

Organoids were fixed in 2.5% glutaraldehyde in 0.01 M PBS, pH 7.2–7.4, for 30 min, harvested in an Εppendorf tube and centrifuged at 1500 × g for 5 min at 4°C forming a pellet. The supernatant was aspirated and organoids were then embedded in warmed 4% gelatin aqueous solution, which was solidified by cooling on ice. Under the stereoscope, the organoid-containing gelatin was extracted from the Eppendorf tube and cut into small fragments (1-2mm^3^). The standard procedure for TEM processing of specimens (gelatin-organoid fragments) was followed, i.e., post-fixation with OsO_4_, dehydration, infiltration, and embedding in epoxy resins. Ultra thin epoxy sections (70-80 nm thickness) were cut using a Diatome diamond knife, were mounted onto 200-mesh copper grids and stained with alcoholic uranyl acetate and lead citrate. Finally, they were observed on a Jeol JEM 2100Plus TEM (Japan) equipped with a OneView digital camera (Gatan, USA).

**Seahorse XFe96 Metabolic Flux Analysis**

Real-time oxygen consumption rates (OCRs) and extracellular acidification rates (ECARs) were determined using the Seahorse Extracellular Flux (XFe96) analyzer (Seahorse Agilent) [17]. Briefly, untreated or piceatannol/tyrphostin 23-treated cells (2 × 10^4^ cells per well) were seeded into XFe96 cell culture plates and incubated for 6 h to allow cell attachment. Cells were subsequently washed in pre-warmed XF assay media (or for OCR measurement, XF assay media supplemented with 10 mM glucose, 1 mM Pyruvate, 2 mM L-glutamine, and adjusted at 7.4 pH). Cells were then maintained in 175 μL/well of XF assay media at 37 °C, in a non-CO_2_ incubator for 1 h. During the incubation time, 25 μL of 80 mM glucose, 9 μM oligomycin, and 500 mM 2-deoxyglucose were loaded for ECAR measurement or 10 μM oligomycin, 9 μM FCCP, 10 μM rotenone, 10 μM antimycin A were loaded for OCR measurement, in XF assay media into the injection ports in the XFe96 sensor cartridge. Measurements were normalized by trypan blue and protein content (SRB assay) [18]. Datasets were analysed with the XFe96 and GraphPad Prism software, using two-way ANOVA and Student’s *t* test calculations. All experiments were performed in quintuplicate, three times independently.

**Hotspot and Whole exome sequencing**

Hotspot sequencing was carried out as previously described [4]. For whole exome sequencing (WES), genomic DNA was extracted from organoid lines using the DNeasy Blood & Tissue Kit (Qiagen). Library construction and sequencing were performed by Macrogen Europe. The sequencing library was prepared by random fragmentation of the DNA, followed by 5' and 3' adapter ligation using the SureSelect V6-Post Library kit. Adapter-ligated fragments were then PCR amplified and gel purified. Paired-end sequencing was carried out on an Illumina platform. Paired-end 151 bp reads were first trimmed with TrimGalore (<https://github.com/FelixKrueger/TrimGalore>) and then aligned to the human genome version GCCh38/hg38 using the Burrow-Wheeler Aligner BWA-MEM [19]. Duplicate reads were removed with Picard tool-MarkDuplicates (<http://broadinstitute.github.io/picard>). SAMtools mpileup and bcftools [20], GATK tools [21] were used for identification and filtering of the SNPs and INDELs. Copy number and structural variants were determined using MANTA [22] and annotation on the human reference genome was performed with ANNOVAR [23]. Genomic distribution was performed using BEDTools [24] and the -closetBed and -intersectBed subcommands, respectively.

**Transcription factor motif scanning**

The *LEP* promoter sequence (plus upstream 2kbps) was scanned for occurrences of known motifs, treating each motif independently using FIMO [25] from MEME-suite. The log-odds scores were converted into p-values, assuming a zero-order background model. All motif occurrences with a *p*-value less than 1e-03 were reported. The *p*-values for each motif occurrence were converted to *q*-values following the Benjamini and Hochberg method. The motif logo sequences were constructed according to their position weight matrices.

**Molecular docking simulations *in silico***

The crystal structures of a HIF1A peptide bound to the VHL interface (pdb id: 6GMR) and VHL in complex with a methylisoxazolyl derivative (pdb id: 3ZRC) were used for all simulations. Docking calculations were performed using the Glide XP algorithm (Schrodinger Inc.) with an extended grid box and a distance-dependent dielectric value set at 4. The scaling factors for non-polar atoms were set to 90% and 80% for protein and ligand atoms, respectively. Partial charges and electrostatic potential of piceatannol and tyrphostin 23 were determined by *ab initio* quantum-mechanical calculations at the Density Functional Theory (DFT) level of theory by use of the B3LYP functional and a 6-311G basis set with full polarization and diffusion functions. Molecular dynamics (MD) simulations were performed by the Desmond software (D. E. Shaw research). Different MD systems were prepared by charge neutralizing and solvating the protein-ligand complexes to TIP3P and SPC/E waters and 0.15 mM NaCl. Periodic boundary conditions were applied to a triclinic system with a buffer range of 1 nm and simulations were performed at the NPT ensemble (Nose-Hoover chain thermostat, Martyna-Tobias-Klein barostat) for 0.5 μsec at 300 K. Molecular dynamics simulations were performed at a Linux mint local workstation operating a 2080 Super GPU chipset. The metadynamics calculations entailed 10 x 10 nsec simulations, with the ligand RMSD from starting coordinates as a collective variable and a subsequent analysis of ligand stability based on the ligand geometry RMSD (PoseScore) and intermolecular hydrogen bond persistence (PersScore) metrics. The hydration calculations were performed by the SZmap algorithm at the stabilisation mode.

***In vivo* mouse experiments**

For toxicity assays, piceatannol (Selleckchem #S3026) and tyrphostin 23 (AdooQ Bioscience #A15940) were administered intraperitoneally and orally, respectively, into NOD.CB17-Prkdc^scid^/NCrHsd mice in a daily dose of 100 mg/Kg of body weight for 14 days. Each drug, resuspended in DMSO (100 mg/mL), was further diluted in Cremophor EL (Millipore #238470) and saline (NaCl 0.9%) to 1 (DMSO): 1 (Cremophor EL): 8 (Saline) ratio to achieve a tolerable dosage regimen for mice (10 mg/mL working solution). 14 days post treatment, blood tests were performed to assess the safety of both drugs compared to the control counterparts. Weight measurements on all mice were carried out every 2 days since the beginning of the treatment.

Human organoid xenotransplantations in NOD.CB17-Prkdc^scid^/NCrHsd mice were carried out as previously described [4]. For orthotopic transplantations, 10^5^ cells were resuspended in 20-50 μL of BME and injected intratracheally into mouse lungs. 5 months later, the mice were sacrificed using a lethal dose of ketamine/xylazine and the lungs were perfused, removed and fixed with 10% formalin to carry out complete histopathological assessments. Stereoscopic images of tumours were acquired on a Stemi DV4 Zeiss stereoscope, on a 32X or 48X Objective. The number of injected cells was estimated using the Disposable Haemocytometer (C-Chip). For subcutaneous injections, 5 x 10^6^ organoids were injected into the flank of the same mouse strain. Approximately 1 month after the injection, upon formation of palpable tumours, piceatannol and tyrphostin 23 were administrated for 14 days as described above. Tumour size was estimated by a digital caliper, as previously described [26], until the end of the treatment when tumours were extracted, fixed in 10% formalin and histologically evaluated. Experiments on NOD-SCID mice were carried out at the National Centre for Scientific Research (NCSR) “Demokritos” according to local and international regulations and ethical guidelines, and were approved by the local animal experimental committee at the NCSR “Demokritos” (Protocol number: 433154).

**Statistical analysis**

Statistical analysis was carried out with the GraphPad Prism software, versions 6 and 7. Two-tailed Student’s t-test analysis was implemented to evaluate the statistical significance of differences between two experimental groups.

**References**

1. Trapnell, C., L. Pachter, and S.L. Salzberg, *TopHat: discovering splice junctions with RNA-Seq.* Bioinformatics, 2009. **25**(9): p. 1105-11.

2. Love, M.I., W. Huber, and S. Anders, *Moderated estimation of fold change and dispersion for RNA-seq data with DESeq2.* Genome Biol, 2014. **15**(12): p. 550.

3. Robinson, M.D., D.J. McCarthy, and G.K. Smyth, *edgeR: a Bioconductor package for differential expression analysis of digital gene expression data.* Bioinformatics, 2010. **26**(1): p. 139-40.

4. Sachs, N., et al., *Long-term expanding human airway organoids for disease modeling.* EMBO J, 2019. **38**(4).

5. Schwank, G., et al., *Functional repair of CFTR by CRISPR/Cas9 in intestinal stem cell organoids of cystic fibrosis patients.* Cell Stem Cell, 2013. **13**(6): p. 653-8.

6. Drost, J., et al., *Sequential cancer mutations in cultured human intestinal stem cells.* Nature, 2015. **521**(7550): p. 43-7.

7. Ran, F.A., et al., *Genome engineering using the CRISPR-Cas9 system.* Nat Protoc, 2013. **8**(11): p. 2281-2308.

8. Katsouda, A., et al., *MPST sulfurtransferase maintains mitochondrial protein import and cellular bioenergetics to attenuate obesity.* J Exp Med, 2022. **219**(7).

9. Katsouda, A., et al., *An optimized protocol for chromatin immunoprecipitation from murine inguinal white adipose tissue.* STAR Protoc, 2023. **4**(4): p. 102594.

10. Papaspyropoulos, A., et al., *RASSF1A uncouples Wnt from Hippo signalling and promotes YAP mediated differentiation via p73.* Nat Commun, 2018. **9**(1): p. 424.

11. Mourkioti, I., et al., *A GATA2-CDC6 axis modulates androgen receptor blockade-induced senescence in prostate cancer.* J Exp Clin Cancer Res, 2023. **42**(1): p. 187.

12. Papaspyropoulos, A., et al., *Decoding of translation-regulating entities reveals heterogeneous translation deficiency patterns in cellular senescence.* Aging Cell, 2023: p. e13893.

13. Lagopati, N., et al., *Biological Effect of Silver-modified Nanostructured Titanium Dioxide in Cancer.* Cancer Genomics Proteomics, 2021. **18**(3 Suppl): p. 425-439.

14. Papaspyropoulos, A., et al., *RASSF1A disrupts the NOTCH signaling axis via SNURF/RNF4-mediated ubiquitination of HES1.* EMBO Rep, 2022. **23**(2): p. e51287.

15. Magkouta, S., et al., *A fluorophore-conjugated reagent enabling rapid detection, isolation and live tracking of senescent cells.* Mol Cell, 2023. **83**(19): p. 3558-3573 e7.

16. Zampetidis, C.P., et al., *A recurrent chromosomal inversion suffices for driving escape from oncogene-induced senescence via subTAD reorganization.* Mol Cell, 2021. **81**(23): p. 4907-4923 e8.

17. Fiorillo, M., et al., *Bedaquiline, an FDA-approved drug, inhibits mitochondrial ATP production and metastasis in vivo, by targeting the gamma subunit (ATP5F1C) of the ATP synthase.* Cell Death Differ, 2021. **28**(9): p. 2797-2817.

18. Vichai, V. and K. Kirtikara, *Sulforhodamine B colorimetric assay for cytotoxicity screening.* Nat Protoc, 2006. **1**(3): p. 1112-6.

19. Li, H. and R. Durbin, *Fast and accurate short read alignment with Burrows-Wheeler transform.* Bioinformatics, 2009. **25**(14): p. 1754-60.

20. Li, H., et al., *The Sequence Alignment/Map format and SAMtools.* Bioinformatics, 2009. **25**(16): p. 2078-9.

21. McKenna, A., et al., *The Genome Analysis Toolkit: a MapReduce framework for analyzing next-generation DNA sequencing data.* Genome Res, 2010. **20**(9): p. 1297-303.

22. Chen, Y.A., et al., *MANTA, an integrative database and analysis platform that relates microbiome and phenotypic data.* PLoS One, 2020. **15**(12): p. e0243609.

23. Wang, K., M. Li, and H. Hakonarson, *ANNOVAR: functional annotation of genetic variants from high-throughput sequencing data.* Nucleic Acids Res, 2010. **38**(16): p. e164.

24. Quinlan, A.R. and I.M. Hall, *BEDTools: a flexible suite of utilities for comparing genomic features.* Bioinformatics, 2010. **26**(6): p. 841-2.

25. Grant, C.E., T.L. Bailey, and W.S. Noble, *FIMO: scanning for occurrences of a given motif.* Bioinformatics, 2011. **27**(7): p. 1017-8.

26. Magkouta, S.F., et al., *MTH1 favors mesothelioma progression and mediates paracrine rescue of bystander endothelium from oxidative damage.* JCI Insight, 2020. **5**(12).
